# Measurement of the Earth's rotation: 720 BC to AD 2015

**DOI:** 10.1098/rspa.2016.0404

**Published:** 2016-12

**Authors:** F. R. Stephenson, L. V. Morrison, C. Y. Hohenkerk

**Affiliations:** 1 University of Durham, Durham, UK; 2 Pevensey, East Sussex, UK; 3 HM Nautical Almanac Office, UK Hydrographic Office, Taunton TA1 2DN, UK

**Keywords:** eclipses, occultations, length of day, tidal friction, core–mantle coupling, sea level

## Abstract

New compilations of records of ancient and medieval eclipses in the period 720 BC to AD 1600, and of lunar occultations of stars in AD 1600–2015, are analysed to investigate variations in the Earth’s rate of rotation. It is found that the rate of rotation departs from uniformity, such that the change in the length of the mean solar day (lod) increases at an average rate of +1.8 ms per century. This is significantly less than the rate predicted on the basis of tidal friction, which is +2.3 ms per century. Besides this linear change in the lod, there are fluctuations about this trend on time scales of decades to centuries. A power spectral density analysis of fluctuations in the range 2–30 years follows a power law with exponent −1.3, and there is evidence of increased power at a period of 6 years. There is some indication of an oscillation in the lod with a period of roughly 1500 years. Our measurements of the Earth’s rotation for the period 720 BC to AD 2015 set firm boundaries for future work on post-glacial rebound and core–mantle coupling which are invoked to explain the departures from tidal friction.

## 1. Introduction

In a previous paper, two of the present authors set out in detail the provenance, content and analysis of historical astronomical observations pertaining to the measurement of long-term fluctuations in the Earth’s rotation [1].

Since that analysis, about 25% more ancient Babylonian eclipse observations have been added to the dataset, and the Chinese observations have been thoroughly revised for previous inaccuracies of interpretation.

The archive of lunar occultations from AD 1623 onwards has been re-reduced by Herald & Gault [2] using the latest Jet Propulsion Laboratory (JPL) lunar ephemerides, star positions and outline of the Moon. These developments significantly improve the reliability of the determination of decade and longer-term fluctuations in the Earth’s rotation in the period 720 BC to AD 2015.

We present our results in graphical and tabular form to assist future investigations of the underlying geophysical mechanisms driving changes in the Earth’s rotation.

Extensive tabular material and subject matter secondary to this investigation are included in the electronic supplementary material to this paper. The numbers of the tables referred to in this paper are prefixed by ‘S’. Electronic supplementary material may be found on this journal’s website at http://rspa.royalsocietypublishing.org/lookup/doi/10.1098/rspa.2016.0404.

### (a) Timescales and ΔT

The independent argument of time in the gravitational theories of motion of Solar System bodies defines a theoretically uniform time scale, which is denoted by Terrestrial Time (TT). The time scale, based on the (variable) rotational period of the Earth, is denoted by Universal Time (UT). The difference (TT−UT) between the recorded times on UT of events, such as eclipses or occultations, and their predicted times on TT using the gravitational theories of their motion, is designated by ΔT. Since 1955.5, highly stable atomic clocks have provided an independent, uniform time scale (TAI), which is related to TT by

1.1 TT=TAI+32.184 s.

The standard unit of time on the TT and TAI scales is the day of 86400 SI seconds exactly. The day on the UT scale is the mean solar day, which is derived from the (variable) period of the Earth’s rotation. Over a selected interval, the quantity ΔT is the cumulative difference in time between the fixed standard day and the variable mean solar day.

### (b) Tidal friction and the behaviour of ΔT

The current value of the tidal acceleration of the Earth’s spin has been measured reliably from the perturbations by lunar and solar tides on near-Earth satellites, together with the requirement that angular momentum be conserved in the Earth–Moon system. This result leads to an empirical relation between the acceleration of the Earth’s spin, ${\dot{\omega }}_{\mathrm{tidal}}$, and the observed orbital tidal acceleration of the Moon, $\dot{n}$ [3]

1.2 $${\dot{\omega }}_{\mathrm{tidal}}=+(49\pm 3)\times 0.004\;869\dot{n}\times 10^{-22}\;{\mathrm{rad}\;s}^{-2}.$$

Lunar laser ranging provides an accurate value for the Moon’s tidal acceleration, −25.82±0.03′′ cy^−2^ [4], and inserting this in the above relation gives the result

1.3 $${\dot{\omega }}_{\mathrm{tidal}}=-6.16\pm 0.4\times 10^{-22}\;{\mathrm{rad}\;s}^{-2}.$$

Thus, ΔT_tidal_ should behave parabolically with time

1.4 $$\Delta T_{\mathrm{tidal}}=ct^{2}\;s,$$

where *t* is measured in Julian centuries from an epoch around 1820, which is where the average length of the mean solar day is equal to the standard of 86400^s^ SI (see Stephenson & Morrison [1]). If the acceleration coefficient *c* is measured in s cy^−2^, then the conversion factor to rad s^−2^ is −1.46×10^−23^ (see Stephenson & Morrison [5, table 2]), and thus

1.5 $$\Delta T_{\mathrm{tidal}}=+(42\pm 2){\left(t-18.2\right)}^{2}\;s.$$

The satellite and lunar laser ranging measurements were obtained from data collected over about 50 years or so. However, they can be applied to the past few millennia because it has been demonstrated satisfactorily that the mechanism of tidal friction has not changed significantly during this period [6].

While the tidal component of the Earth’s acceleration can be derived from recent high-precision observations, the actual long-term acceleration, which is the sum of the tidal and other components, cannot be measured directly from modern data because it is masked by the relatively large decade fluctuations. Instead, observations from the historical past, albeit crude by modern standards, have to be used. By far the most accurate data for measuring the Earth’s rotation before the advent of telescopic observations (*ca* AD 1620) are records of eclipses. Useful records of eclipses extend back to about 720 BC. After AD 1620, and before the introduction of atomic time, timings of lunar occultations of stars provide the most accurate data on the decade behaviour of the Earth’s rotation.

## 2. Observations: description and provenance

The observations of eclipses fall into two broad categories. First, eclipse phenomena that were timed in a local system that permits the UT of the observations to be recovered. Second, observations that were not timed, but where the configuration of the eclipses allows the fixing of the Earth’s rotational frame, and hence the UT. We treat these two groups separately until the analysis stage, where they are combined. In the mathematical analysis of the data and in the tables, we use the continuous algebraic notation for years, rather than the discontinuous BC/AD scale which has no year zero. Negative dates differ by 1 from BC dates (e.g. −708 is equivalent to 709 BC). A plus sign is to be understood where we do not give a sign before the year.

### (a) Timed observations

The evaluation of expression (1.5) for *t*=−7 produces a value of ΔT_tidal_ of over 7 h. Thus, ancient observations of solar and lunar eclipses, many of which were timed to a fraction of an hour, have the potential to enable changes in the Earth’s rate of rotation to be determined. We have systematically used data from a wide variety of cultures. These include observations made in Babylon, China and Greece. During the medieval period, the observations were mainly made in the Arab Dominions and China; it should be noted that there are scarcely any medieval European measurements of eclipse times.

After the introduction of the telescope in the seventeenth century, the instants of lunar occultations of stars, given nominally to a precision of 1 *s* or better, provide the most accurate measurement of ΔT until the introduction of the atomic time scale, TAI. Although the given precision is 1 *s*, the accuracy is certainly poorer than this in the seventeenth century. Timing of lunar occultations, which continue to this day, have been collected and collated in the past by several astronomical institutions, and the largest compilation, comprising many thousands of observations, is now archived in the Centre de Données Astronomiques de Strasbourg (CDS) [2].

#### (i) Babylon

Over a period of several centuries—from the eighth century BC until perhaps well into the first century AD—Babylonian astronomers systematically measured the times of the beginning, end and often maximum of eclipses (in addition to observing other lunar and planetary events). The Babylonian scribes wrote on clay tablets, using a cuneiform script, which is now well understood. It just so happens that, because there are so many broken texts, we often have only the time of first contact. One of their motives for observation was to improve prediction of future eclipses, and many predictions (usually of the time of onset of an eclipse) are cited in the astronomical texts.

Nearly all of the known astronomical texts—most of which are in a fragmentary condition—were acquired by the British Museum in the late nineteenth century and some 2000 texts devoted to astronomy are now carefully preserved there. It is estimated that fewer than 10% of the original records are now extant. Most of the surviving eclipse texts are of lunar eclipses; these range in date from some time before 700 BC to around 50 BC. However, no solar eclipse records are known prior to about 350 BC, whereas the latest known report probably dates from 10 BC. The existence of several later Babylonian almanacs—the latest dating from AD 75—suggests that regular observations continued until well into the first century AD, although no observational texts survive from this period.

In early centuries, the Babylonians numbered years from the start of the reign of each king. However, commencing in 311 BC (the Seleucid era), a continuous scheme of numbering years was adopted. The operation of the Babylonian luni-solar calendar has been extensively studied, and tables are available for conversion of dates to the Julian calendar [7].

Although many Babylonian texts are fragmentary, the date is often preserved. If a text is damaged and the date is either incomplete or missing, then it is frequently possible to restore the date. For instance, some texts assemble the records of eclipses at 18 year intervals, and because several dates in each series are sometimes recorded, it is often possible to reliably restore individual dates. In other examples, such as astronomical diaries, there are planetary and lunar observations in addition to eclipses. By retrospective computation of the planetary and lunar data, a unique date can often be derived for a text. It should be emphasized that, in our investigation, we have specifically avoided using any isolated observations of eclipses in which a damaged or missing date has been restored on the basis of the eclipse record itself. Because a preliminary value of ΔT needs to be assumed as an aid to dating, this amounts to circular reasoning. For instance, we have not used a Babylonian observation dated to 10 BC by Steele [8].

Since our previous paper was published [1], we have included many additional data made available by Hunger [9,10] in his extensive transliteration and translation of the extant texts. We have analysed over 180 Babylonian eclipse timings. The usual method of timing followed by the Babylonian astronomers was to measure with some form of clock (probably a clepsydra) the time of the appropriate eclipse phenomenon in relation to sunrise or sunset. After about 560 BC, all time intervals were quoted to the nearest *US* (‘degree’, equal to 4 min). However, in earlier years, time intervals were usually expressed only to the nearest 5^°^ or even 10^°^. After about 250 BC, an additional method of timing lunar eclipses was introduced: relative to the culmination of a selected star or star group. These various timings—whether measured relative to sunrise, sunset or stellar culmination—can be readily converted to local apparent time and hence to UT.

Each month began with the first visibility of the new crescent Moon. We have adopted the definition of sunrise or sunset as the moment when the upper limb of the Sun is on the visible horizon, allowing for a mean refraction of 34′. For details on the methods of timing used by the Babylonian astronomers, the reader is referred to Stephenson & Morrison [1]. The Julian dates of the observations, together with some computational details, are displayed in the electronic supplementary material, tables S1–S3.

Several further Babylonian lunar eclipse timings, ranging in date from 721 BC to 382 BC, are cited by the great Greek astronomer Ptolemy (*ca* AD 150) in his *Mathematike Syntaxis*, which later became known as the *Almagest*. Ptolemy reports that several of these observations had been acquired by Hipparchus of Rhodes in the late second century BC. The Babylonian timings as cited in the *Almagest* were all converted from sunrise or sunset measurements in *US* to the Greek system of fractions of equinoctial and seasonal hours (see §2a(iii)). None of the Babylonian observations recorded by Ptolemy survive in their original form. Hence, it is not possible to assess *a priori* the accuracy with which the Babylonian measurements were reduced to their Greek equivalents. However, we have retained the observations for analysis as a separate set; they are displayed in the electronic supplementary material, table S4.

#### (ii) China

The principal sources of eclipse observations in Chinese history are the official dynastic histories. Most of these histories, which were typically compiled within about a century or two after the fall of a dynasty, have a special section devoted to astronomical records. An additional important source is the *Wenxian Tongkao* (‘Comprehensive study of documents and records’), compiled by Ma Duanlin around AD 1300. A useful supplementary source of observations is the *Zhongguo Gudai Tianxiang Jilu Zongji* (‘A union table of ancient Chinese records of celestial phenomena’) published by Beijing Observatory in 1988.

From ancient times until the early twentieth century, the Chinese calendar was luni-solar. In early centuries, years were numbered from the start of a ruler’s reign. However, from the second century BC onwards, years were also counted from the start of a reign period (a subdivision of an emperor’s reign). Most years contained 12 lunar months, each of 29 or 30 days, with an occasional 13th (intercalary) month—usually every 30 months or so—to keep the calendar in step with the seasons. Solar eclipses almost invariably occurred on the first or last day of a lunar month. The Chinese calendar has been extensively studied by historians, and concordances between years on the Chinese calendar and their Julian or Gregorian equivalent are readily available [11]. Well before 1000 BC, a 60 day cycle was introduced and its use continued without interruption until modern times. The use of this regular cycle, independent of any astronomical parameter, greatly facilitates accurate conversion of dates to the Western calendar. (Based on the tables of Hsueh & Ou-yang [11], one of the present authors (F.R.S.) has developed a computer program to expedite date conversion. A copy can be obtained from him.) Beginning with the *Chunqiu* period (722–481 BC), virtually all Chinese eclipse dates are accurately reported. The dates of all but four of the 37 solar eclipses recorded in the *Chunqiu* prove to be exactly correct [12]. Comparable precision in dating continues throughout later Chinese history. The Chinese calendar was adopted in both Korea and Japan at a fairly early period, the main difference being that years were expressed in terms of the reigns of the appropriate rulers (kings in Korea, emperors in Japan), rather than Chinese monarchs.

No Korean timings of eclipses are preserved until recent centuries. Commencing in AD 1021, several timings of eclipses are preserved in Japanese history [13]. However, in most cases, it is difficult to decide whether the recorded times were based on observation or were calculated by Japanese astronomers. Hence, we shall not consider the Japanese timed data further.

Measurements of eclipse times are by no means systematically preserved in Chinese history. No careful measurements are extant prior to as late as AD 434. Between this date and AD 768, the measured times of some 20 solar and lunar eclipses are recorded. However, this reasonably productive period is followed by a significant lacuna in which virtually no similar observations are preserved in Chinese history until AD 1050. Once again, fairly regular solar and lunar eclipse timings are extant from this date until AD 1280, but over the next three centuries records are very sporadic.

We have terminated our investigation at AD 1280. By then, the value of ΔT has become so small (less than about 10 min) that the available measurements fail to be of use in studying changes in Earth’s past rotation. In this investigation, we have introduced only a few hitherto unused measurements of eclipse times from China. However, we have paid special attention to the translation of several eclipse reports whose interpretation had seemed doubtful, based on help from sinologist colleagues.

In general, Chinese solar and lunar eclipse records make no mention of the time of day or night. However, the astronomers frequently timed solar eclipses to the nearest *ke* (‘mark’), equal to 1/100th of a day or 0.24 h. Prior to around AD 1050, nearly all lunar eclipses were timed to the nearest fifth of a night watch. Because the night was divided into five watches (*geng*), the corresponding precision of measurement was relatively low, averaging approximately 0.5 h. After about AD 1050, the precision of timing lunar eclipses improved; most measurements were then quoted to the nearest *ke*, in common with solar eclipses.

The dates of the observations are collected in the electronic supplementary material, tables S5 (lunar) and S6 (solar).

#### (iii) Ancient Greece

Only eight timings of lunar eclipses observed in ancient Greece are preserved; these are all cited in Ptolemy’s *Almagest*, and mainly range in date from 201 BC to 141 BC. A single further observation dates from AD 125. The accuracy with which the measured times are quoted is typically to the nearest third of an hour. After AD 125, no further eclipse timings are preserved from ancient Greece—or elsewhere in Europe—until AD 364. In this year, Theon of Alexandria timed the beginning, middle and end of a partial solar eclipse to the nearest fifth or sixth of a seasonal hour [14]. In modern times, this important set of observations first attracted the attention of Fotheringham [15]. The dates of the observations are collected in the electronic supplementary material, table S7.

#### (iv) Medieval Europe

After the solar eclipse times reported by Theon of Alexandria in AD 364, no further European measurements of eclipse times are known to be preserved for nearly 1000 years—until the mid-thirteenth century [16]. However, by then, ΔT was so small that the few preserved observations are too inaccurate to prove of value.

#### (v) Arab dominions

Extant Arab observations of eclipse times can be found in treatises on astronomy by three medieval astronomers: Ibn Yunus, al-Battani and al-Biruni. The various records were translated and studied in detail by Said & Stephenson [17]. The observations were mainly made at observatories in Baghdad and Cairo. In total, more than 50 measurements are preserved, all from the period about AD 830 to 1020. In particular, the measurements recorded by Ibn Yunus, who died in AD 1009, survive only in a single manuscript written by his son. This important manuscript, preserved in Leiden University Library, contains some very careful observations, several made by Ibn Yunus himself.

The dates of eclipses (and other celestial phenomena) recorded in the Islamic world number years from al-Hijrah, the migration of the Prophet Muhammad from Mecca to Medina in AD 622. All years on the Islamic calendar consist of 12 lunar months, each of either 29 or 30 days. The start of each month is determined by the first sighting of the new crescent Moon. Intercalation has never been practised, so that the Muslim year is about 11 days shorter than the solar year. This results in gradual regression of the start of the year—and in particular the beginning of the ninth month Ramadan—through the seasons in about 33 years. Because sighting of the lunar crescent is affected by atmospheric conditions, historians usually effect date conversion to the Julian or Gregorian calendar using tables based on a ‘standardized’ Islamic calendar with alternate 30 and 29 day months [18]. When the dates of eclipses and other astronomical events, whether noted in treatises on astronomy or in chronicles, are converted to the Western calendar there is usually exact accord with the dates derived by modern astronomical computation.

Medieval Muslim astronomers mainly measured the local times of eclipse contacts indirectly, by determining the altitude of the Sun (for solar eclipses) or the altitude of the Moon or a selected bright star (for lunar eclipses). These measurements were then reduced to local times using astrolabes. Most altitudes were quoted to the nearest degree. The precision in time is thus equivalent to about 4 min. No ‘new’ Arab measurements have come to light since the work of Stephenson & Morrison [1]. However, we have now re-analysed each of the Arab observations in order to eliminate possible computational errors, which may have occurred in our previous publications.

The dates of the observations are collected in the electronic supplementary material, table S8 (solar) and table S9 (lunar).

#### (vi) Telescopic observations after AD 1600

Until the introduction of TAI, timings of dark limb lunar occultations of stars provide the most accurate measure of ΔT. Thousands of occultation timings starting with AD 1623 have been assembled, collated and analysed for this purpose by various authors. The latest collation of about half a million observations is that assembled by Herald & Gault [2]. Besides the reported date and time of observation in UT, each record gives the method of timing, the estimated accuracy and various parameters associated with the reduction of the observation.

The number of observations increases roughly exponentially from the seventeenth century onwards.

#### (vii) Atomic time data

The values of TAI−UT—specifically TAI−UT1—are tabulated by the International Earth Rotation & Reference Systems Service (IERS) [19] at a daily interval from 1962 to the present.

We have only used the TAI dataset 1962–2015 as a comparative control on the smoothing of the occultation dataset, and have not substituted them for the occultations.

### (b) Untimed observations

Untimed reports of total solar eclipses recorded at specific places can be used to set precise limits on the value of ΔT at discrete dates. Owing to the narrowness of the path of totality on the Earth’s surface, a report that an eclipse was total or near-total at a known place fixes the rotational frame of the Earth to within the projected width of the band of totality parallel to the Earth’s equator. This width is usually only a few minutes of time, and thus an untimed total eclipse can provide useful limits on the value of ΔT at the epoch of the eclipse, provided we know the date of the eclipse and where it was observed. We denote these limits by the terms upper and lower bounds of the solution space for ΔT, which has a rectangular probability distribution function between these bounds.

Untimed observations of large partial solar eclipses can also set useful limits to ΔT. Many early records—both by astronomers and by untrained observers in various parts of the world—cite untimed observations of solar eclipses in which a small portion of the Sun remained visible at maximal phase. The contrast between a total eclipse and one that fell only slightly short of totality is so impressive that even an untrained observer, in describing a large partial eclipse, can provide a valuable report. An observation specifically asserting the occurrence of a large partial solar eclipse defines a pair of limits to the value of ΔT, between which lies an excluded zone (that would correspond to totality at the place of observation).

Thus, the solution space for ΔT has a lower bound at the upper boundary of totality and an upper bound at the lower boundary of totality. As the penumbra of an eclipse is wide, the solution space for ΔT extends considerably beyond these limits. The visual impact of a large partial solar eclipse drops off quickly on either side of totality. So, the solution space for ΔT can be regarded as a tapering probability distribution function, with greatest likelihood of the observation being just outside the belt of totality. However, the steepness of this tapering function is dependent on the angle of the eclipse path relative to the Earth’s equator. In the extreme case, where the path is parallel to the equator, the probability function for the solution space for ΔT is flat.

An example of an untimed large partial solar eclipse is as follows. On AD 1330 July 16, the chronicle of the monastery of Aula Regia (now known as Zbraslav) recorded that ‘the Sun was so greatly obscured that of its great body only a small extremity like a three-night old Moon was seen’. We compute that for the eclipse to have been partial at Zbraslav either ΔT<900^s^ or >1220^s^. For intermediate values of ΔT, the eclipse would have been fully total at Zbraslav—in marked discord with the observer’s report.

#### (i) Babylon

Only two observations of a total solar eclipse have come down to us from Babylon: those of 136 BC (−135) and 10 BC (−9). The date of the former observation, which incontestably describes a total solar eclipse, is clearly specified in the text (see electronic supplementary material, section S3, −135) and the report provides an independent estimate of ΔT, whereas the date of the latter event is not preserved, and the derived date is based on circular reasoning—a preliminary value of ΔT having been used to identify the eclipse. This does not meet our usual selection criteria, and therefore has not been used here in fitting curves to ΔT. Nevertheless, we have included it in the electronic supplementary material, table S10.

Several observations by Babylonian astronomers note that the Sun or Moon rose or set while eclipsed. In this context, Babylon proves to be an ideal observing site: it lies in a flat plain and has an extremely level horizon in all directions; hence, there is no need for corrections for horizon profile. These observations fall into two main categories:

(i) Where it is merely stated that the Sun or Moon was visibly eclipsed at rising or setting: this gives a flat probability distribution function for ΔT, starting from one firm boundary defined by the limiting case where the eclipse started or ended on the horizon. On more than 20 occasions between 702 BC and 66 BC, the Moon was said to rise or set eclipsed. However, only a few of these are critical, most having very wide limits for ΔT. We have retained only the more critical observations. These are listed in the electronic supplementary material, tables S12 (solar) and S13 (lunar).
(ii) Where the fraction of the solar or lunar disc obscured is estimated when the luminary was on the horizon: this enables a specific value for ΔT to be deduced, as in the case of a timed eclipse observation. These are listed in the electronic supplementary material, table S14.

Babylonian untimed lunar eclipses in the *Almagest* are included in the electronic supplementary material, table S4.

#### (ii) China

The earliest direct record of a total solar eclipse in the history of any civilization is described in a Chinese state chronicle: the *Chunqiu* (*Spring and Autumn Annals*). It states: ‘Third year of Duke Huan (prince of Lu State), 7th lunar month, day renchen [cyclical day 29], the Sun was eclipsed and it was total’. The date corresponds to 709 BC July 17 on the Julian calendar; and, as retrospective computation reveals, there was indeed a total solar eclipse on this very day.

The untimed solar eclipse observations used in this analysis are listed in the electronic supplementary material, tables S10 (total) and S11 (large partial).

#### (iii) Arab dominions

Between about AD 800 and 1500, Medieval Arab chroniclers recorded several very large eclipses of the Sun. However, only occasionally can we be sure that an eclipse was fully total at a specific site: notably, at Cordoba in AD 912, Baghdad in 1061 and Cizre in 1176. All three observations lead to useful—but by no means critical—values of ΔT. In addition, a detailed account of an annular solar eclipse in AD 873 is preserved. At Nishapur, ‘the Moon’s body was in the middle of the Sun’s body. The light from the remaining portion of the Sun surrounded it (the Moon)’. Regrettably, this careful observation is satisfied by a wide range of values of ΔT.

Ibn Yunus observed in Cairo the very large solar eclipse of AD 1004. He measured the times (from solar altitudes) of the various stages, and he described the greatest phase as follows: ‘The Sun was eclipsed until what remained of it resembled the crescent Moon on the first night of the month’.

This was partial for values of ΔT outside the limits of totality/annularity (1760^s^ to 1920^s^). Unfortunately, we do not know whether the eclipse was partial to the north or south of Cairo.

The untimed observations are listed in the electronic supplementary material, table S10 (total) and S11 (large partial).

#### (iv) Ancient and medieval Europe

Although numerous reports of large solar eclipses are preserved from ancient Europe, in many cases either the date or place of observation is uncertain or it is not clear from the text whether or not the eclipse was fully total. We have felt able to use only two records in this category. The earliest of these is a generally annular eclipse which the Greek historian Thucydides probably himself witnessed at Athens in 431 BC. Thucydides, in his *History of the Peloponnesian War* (II, 28), reported that the Sun ‘assumed the form of a crescent’. A further annular eclipse that was also seen as partial was observed by the Spartan king Agesilaus and his army near Chaeroneia in 394 BC. The historian Xenophon, who was with the army, recorded that ‘the Sun seemed to appear in a crescent shape’ (Hellenica, IV). Both these observations set fairly useful limits to ΔT.

With only rare exceptions medieval European observations of eclipses which prove of value in studying Earth’s past rotation are to be found in monastic and town chronicles. These mainly range in date from about AD 800 to 1500. Although most annalists had little interest in astronomy, they often set on record eclipses and other major celestial events such as comets because of their spectacular nature. No accurate timings of eclipses are reported in these works, but there are many vivid accounts of total and very large solar eclipses. Some of these describe the complete disappearance of the Sun, as well as mentioning the onset of darkness and the appearance of stars. Other solar eclipse reports specifically deny totality, noting that the Sun resembled a crescent. Fortunately, very many Medieval European chronicles have been published in their original language (usually Latin) in extensive compilations such as *Scriptores Rerum Italicarum* and *Monumenta Germaniae Historica*.

Most years are given in terms of Anno Domini (AD) following the convention popularized by the Venerable Bede in the eighth century. Months follow the system adopted in the Julian calendar, together with occasional use of the Kalends, Nones and Ides. The Kalends were always the first day of a month. In every month except March, May, July and October, the Nones occurred on the fifth day and the Ides on the 13th day. However, in these four months just mentioned, both the Nones and Ides took place 2 days later—respectively on the 7th and 15th days of the month. Frequently, the day of the week is also specified. With only rare exceptions, the dates of eclipses cited in medieval European chronicles usually prove to be exactly correct.

These observations are included in the electronic supplementary material, tables S10 and S11.

## 3. Computation of ΔT

### (a) Lunar ephemeris

We have developed our own software for the computation of the contact times on the TT time scale for solar and lunar eclipses. This software was developed from the procedures set out in chapter 8 of the *Explanatory Supplement to The Astronomical Almanac* [20], and has been tested against independent authorities.

The most important parameter in the Moon’s ephemeris from the standpoint of this analysis is the value of its orbital tidal acceleration. The value implicit in the JPL ephemerides DE430, which was used to reduce the occultation observations after AD 1600, is −25.82′′ cy^−2^ [4], This is close to the value of −26.00′′ cy^−2^ introduced to the analytical ephemeris *j*=2 (*Explanatory Supplement* [20]) by us, and used here and in previous work [1,5] in the reduction of the pre-telescopic observations.

**Figure 1.**
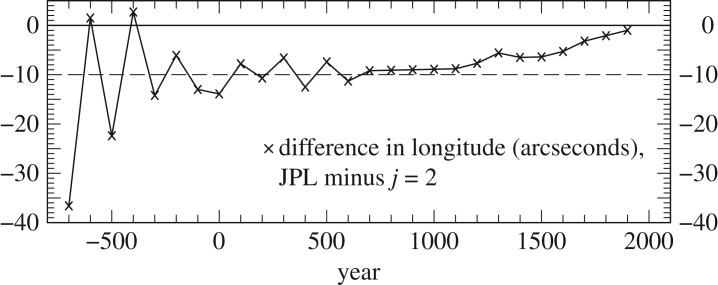
Difference in longitude between JPL lunar ephemeris DE430 and the analytical ephemeris designated *j*=2 incorporating a value of −26′′ cy^−2^ for the Moon’s tidal acceleration. The dashed line is the average difference of about 10′′ in longitude, which produces a systematic difference of about 20^s^ in ΔT.

We have investigated the effect on ΔT of using slightly different values of the lunar tidal acceleration before and after AD 1600. We compared the latest JPL longitudes of the Moon at epochs back to 700 BC with the analytical theory *j*=2, incorporating a tidal acceleration of −26.00′′ cy^−2^. The differences in longitude, employing the same value of the precession constant, are plotted in figure 1. They are negligible in the medieval and ancient period compared to the precision of the observations. This is not the case after AD 1600, where the systematic difference ranges from about +2′′ at AD 1700 to zero at AD 1900, in the sense DE430 − (*j*=2). These account for the systematic differences in ΔT after AD 1600 between this analysis (see §4) and previous work by Stephenson & Morrison [5].

The values of ΔT derived in this paper should be used in conjunction with the lunar ephemeris JPL DE430, or with other ephemerides in which the tidal acceleration is close to the value of −25.82′′ cy^−2^ implicit in the JPL ephemeris.

### (b) Timed observations

We have retained the same scale in the plots of the ancient and medieval results for ΔT, so that the reader can readily judge their relative accuracy.

#### (i) Babylon

In this paper, 180 timings of lunar and solar eclipses in the years −720 to −9 are analysed for the determination of ΔT. The results are listed in the electronic supplementary material, tables S1–S4. In the various texts, it is standard practice for the time of the first contact of an eclipse to be expressed relative to sunrise or sunset, whereas the times of subsequent phases are given after first contact. Random and systematic errors are involved in the observations. The systematic error in the measured time between the observation and the starting or stopping of the clepsydra at sunset or sunrise is likely to be roughly proportional to any systematic drift in the going rate of the clepsydra. Huber & De Meis [21] studied the contribution of these errors, and arrived at an expression for the standard deviation *σ*,

3.1 $$\sigma^{2}=2.8^{2}+{\left(0.14\;d\right)}^{2},$$

where *d* is the elapsed time in degrees from sunset/sunrise to the eclipse contact or vice versa. We have used this variance to weight the observations. In the case of the observations that were timed with respect to the culmination of ziqpu stars, *d* is unknown. Although it was rare for ziqpu star time intervals to exceed 5^°^, the moment of culmination of a star group would be less well defined than sunrise or sunset. The ΔT values were assigned weight 3 by comparison of their dispersion with the other observations.

**Figure 2.**
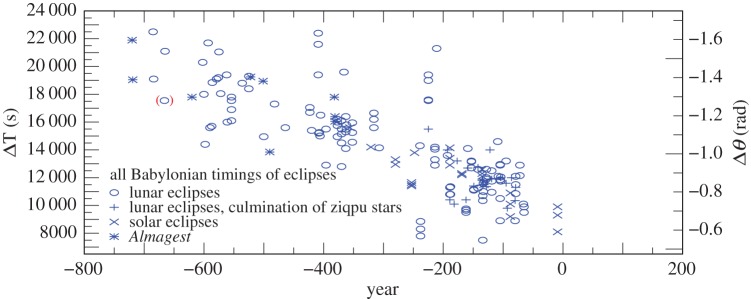
Values of ΔT for all the Babylonian-timed observations −720 to −9 listed in the electronic supplementary material, tables S1–S4. (The conversion factor for deriving the sidereal rotational displacement angle of the Greenwich meridian, Δ*θ*, measured in radians, from ΔT, measured in seconds of mean solar time, is −7.29×10^−5^ ΔT.) The observation in parenthesis was not used in fitting curves to the data (see electronic supplementary material section S4, −666).

**Figure 3.**
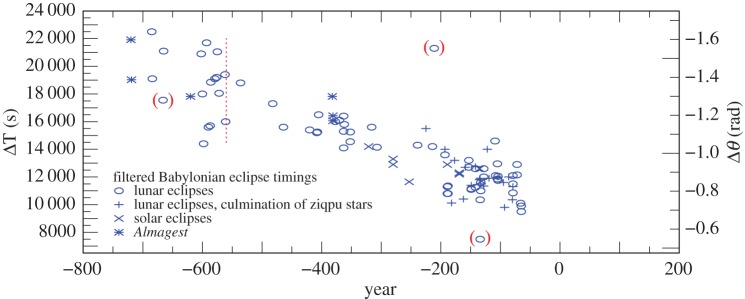
Babylonian observations −720 to −65 with weights greater than 2 after −560, which is indicated by a vertical dotted line. The observations before −560 are intrinsically less accurate (see §3b(i)). The two observations in brackets around −200 were treated as outliers: an observation at −666 is intrinsically doubtful.

Plots of the results for ΔT in the various categories of observation are shown in figure 2. By eliminating observations with the lowest weights (1 and 2) after −560, the dispersion is reduced considerably (figure 3). As stated in §2a(i), the earliest observations (before −560) were only expressed to the nearest 5^°^ or even 10^°^, and weighting these by elapsed time is no longer relevant. The standard deviation (*σ*) of the observations after −560 in figure 3 is 16 min, corresponding to about 4^°^, which is four times the unit of measurement (1^°^). This is probably owing to the difficulty of detecting the precise time of the beginning and ending of eclipses under different observing conditions. The considerably greater *σ* of about 7^°^ for all the observations in figure 2 is dominated by the clock errors that are inherent in timing the longer intervals between sunset/sunrise and the eclipse phenomena.

We investigated the comparative accuracy of lunar eclipses timed with respect to sunset (denoted by + degrees in the electronic supplementary material, table S1) and timed before sunrise (− degrees). This is discussed in the electronic supplementary material, section S2.

The fitting of the finally adopted smooth curve is limited to the observations plotted in figure 3.

#### (ii) China

**Figure 4.**
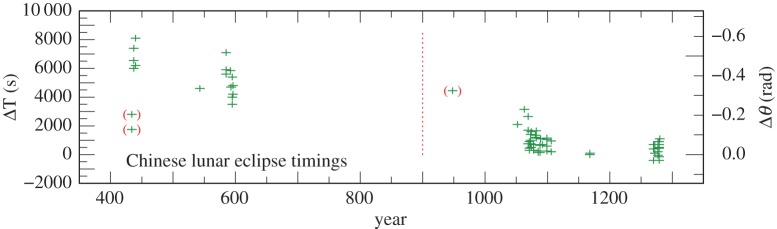
Values of ΔT for Chinese lunar eclipse timings 434–1280 listed in the electronic supplementary material, table S5. The observations are less accurate before 900 (indicated by a vertical dotted line). Observations in brackets were treated as outliers.

**Figure 5.**
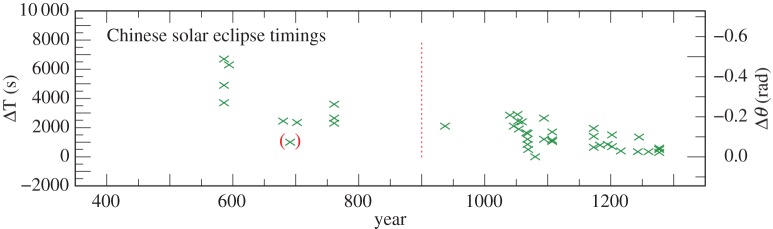
Values of ΔT for Chinese solar eclipse timings 586–1277 listed in the electronic supplementary material, table S6. The observations are less accurate before 900 (indicated by a vertical dotted line). The observation in brackets was treated as an outlier.

We have used records of 111 timings of solar and lunar eclipses in the years 434–1280, which contain sufficiently reliable information to derive values of ΔT. The results are collected in the electronic supplementary material, tables S5, S6, and plotted in figures 4 and 5. Treating the two observations in 434 and those in 691 and 948 as outliers, the standard deviation of the solar observations is 20 min, and the lunar observations 16 min. This difference in accuracy is not particularly significant, and we have not been able to isolate a more accurate subset. All the Chinese observations, apart from the four outliers, have been retained and assigned weight 1 before 900 and 2 thereafter because of their smaller dispersion.

#### (iii) Ancient Greece

**Figure 6.**
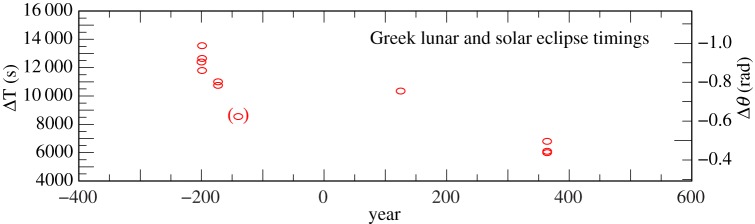
Values of ΔT for Greek lunar and solar eclipse timings −200 to 364, listed in the electronic supplementary material, table S7. The observation in brackets was treated as an outlier.

We have used 11 reliable timings of solar and lunar eclipses recorded in ancient Greece between −200 and +364. The results for ΔT are listed in the electronic supplementary material, table S7, and plotted in figure 6. After rejecting one outlier at −140, the standard deviation is about 14 min, which puts them on a par with the contemporaneous Babylonian observations. They have been assigned weight 3.

#### (iv) Arab dominions

**Figure 7.**
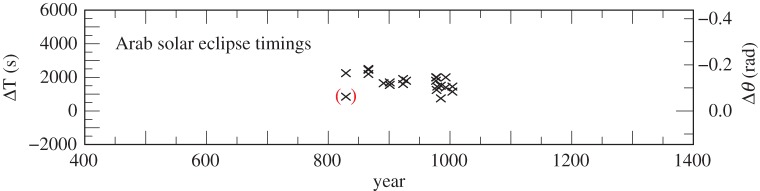
Values of ΔT for Arab solar eclipse timings 829–1004 listed in the electronic supplementary material, table S8. The observation in brackets was treated as an outlier.

**Figure 8.**
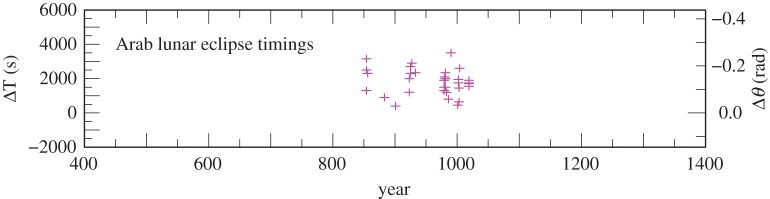
Values of ΔT for Arab lunar eclipse timings 854–1019 listed in the electronic supplementary material, table S9.

The values of ΔT for the Arab timings of solar (22 observations) and lunar eclipses (32 observations) are listed in the electronic supplementary material, tables S8 and S9, and plotted in figures 7 and 8. Their standard deviations from a preliminary curve, after the elimination of one outlier at 829, are 5 min (solar) and 13 min (lunar). The solar timings are the most accurate of the pre-telescopic observations, and have been assigned weight 4. The considerably less accurate lunar observations, which are concurrent with the solar observations, have been omitted in the final fit to the data.

#### (v) Medieval Europe

No timed observations from medieval European sources were used before 1600.

### (c) Collected timed results for ΔT before 1600

**Figure 9.**
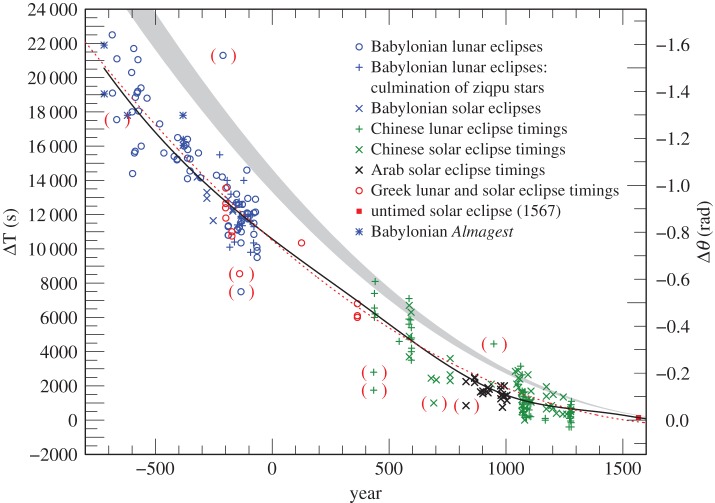
Results for ΔT for collected timed observations −720 to 1280 and the untimed total solar eclipse of 1567. The dotted red curve is the parabola given by equation (4.1). The black curve is the spline curve described in §4b.The grey curve is the parabola (equation (1.5)), predicted on the basis of tidal friction. The observations in brackets were treated as outliers, apart from a Babylonian observation in −666 which is intrinsically doubtful.

The discrete values of ΔT, derived from timed observations in §3b, are plotted in figure 9.

### (d) Telescopic observations after 1600

Herald & Gault [2] have used the latest star positions, lunar ephemeris JPL DE430 and configuration of the Moon’s outline in reducing each observation to obtain the residual separation in arcseconds of the star from the lunar limb (Δ*σ*), reckoned positive outside the limb, assuming an estimate of ΔT derived from various expressions. These initial estimates of ΔT are tabulated or given as functions of time in the ReadMe accompanying the CDS [2] file of observations. Each reduction also includes the change in Δ*σ* owing to a change of +1^s^ in the time of observation, which we refer to as the rate of change. The actual value of ΔT for each observation can thus be recovered from the estimate used in the reduction by subtracting from it Δ*σ* divided by its rate of change. If the occultation is nearly tangential to the motion of the Moon, then the rate of change of Δ*σ* tends to zero, and thus the correction to the estimate of ΔT becomes indeterminate. For this reason, we have used only observations with a rate of change greater than 0.2′′ s^−1^.

After 1945, most of the observational records contain estimates of their accuracy, and many observations are better than the average visual accuracy of ±0.3 s. Before 1945, they do not generally have estimates, and an average error of ±0.3 s has been assumed, if an estimate is not given. As there are many observations after 1945, we retained only observations timed using some electronic device, such as photoelectric equipment, and visual observations with a stated accuracy of ±0.1 s.

The observations before 1700 show considerable scatter, which is due to the difficulty for observers at that epoch in determining UT or its equivalent. We have supplemented the occultation observations with timings of the fourth contacts of about 140 solar eclipses in the period 1623–1670 published by Morrison *et al.* [22]. Although these are not sharp events such as occultations, the resultant error is comparable.

A preliminary curve was fitted to the observations, and residuals from that curve were calculated. The following filters were applied to remove outliers:

$$\begin{array}{cc} 1600-1700: & \pm 100^{\text{s}}\\ 1700-2015: & \pm 25^{\text{s}} \end{array}$$

**Figure 10.**
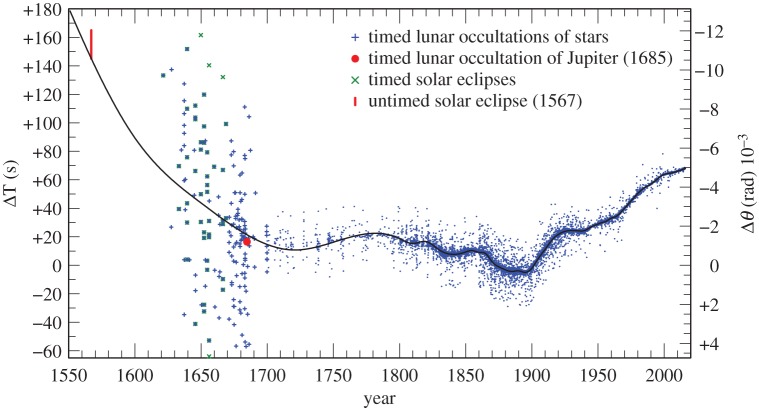
Results for ΔT for timed data 1623–2015: lunar occultations of stars (478 843 observations); lunar occultation of Jupiter;fourth contacts of solar eclipses (1623–1670). The untimed total solar eclipse of 1567 is also plotted. A sample of up to 10 observations in any one year are plotted to avoid saturation, which otherwise would give a false impression of the scatter of the data. The black curve is the spline curve described in §4b.

The values of ΔT derived from occultations and eclipses in the period 1623–2015 which were retained for further analysis are plotted in figure 10.

### (e) Collected untimed results for ΔT

**Figure 11.**
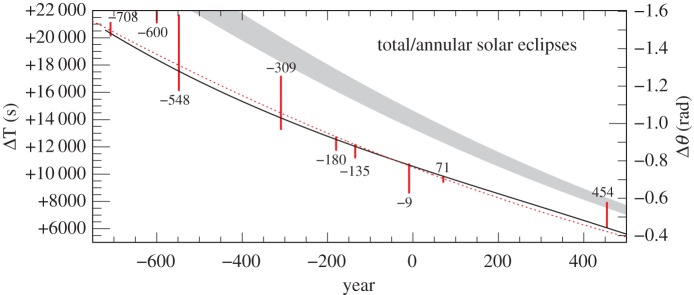
Solution space for ΔT: total/annular eclipses −708 to 454. The dotted red curve is the parabola given by equation (4.1).The black curve is the spline curve described in §4b. The grey curve is the parabola (equation (1.5)), predicted on the basis of tidal friction.

**Figure 12.**
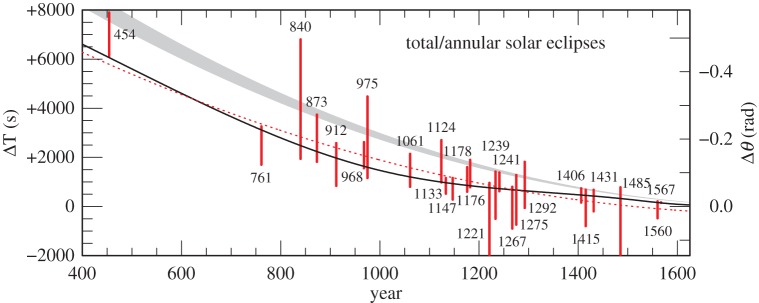
Solution space for ΔT: total/annular eclipses 454–1567. The dotted red curve is the parabola given by equation (4.1). The black curve is the spline curve described in §4b.The grey curve is the parabola (equation (1.5)), predicted on the basis of tidal friction.

**Figure 13.**
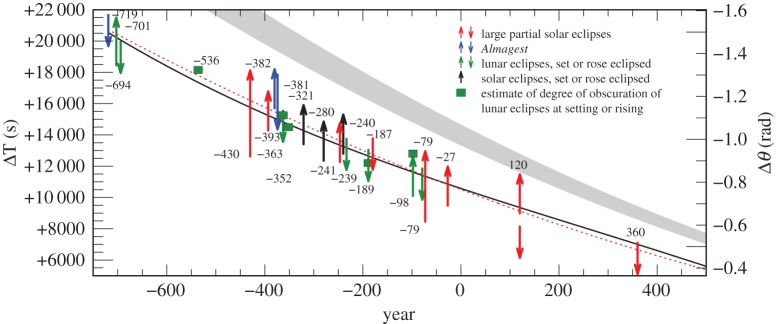
Solution space for ΔT: −719 to 360, large partial solar eclipses; solar and lunar eclipses rose or set eclipsed; estimates of degree of obscuration of lunar and solar eclipses at rising or setting. The dotted red curve is the parabola given by equation (4.1). The black curve is the spline curve described in §4b.The grey curve is the parabola (equation (1.5)), predicted on the basis of tidal friction.

**Figure 14.**
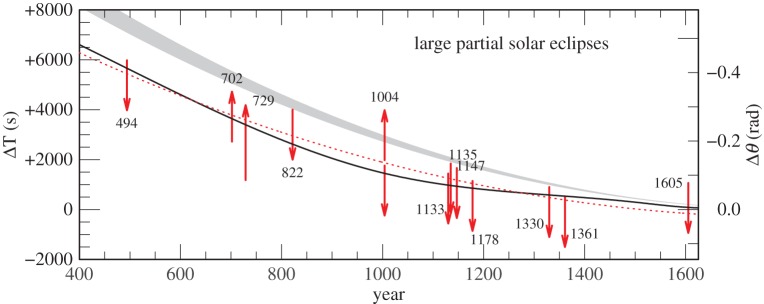
Solution space for ΔT: the dotted red curve is the parabola given by equation (4.1). The black curve is the spline curve described in §4b.The grey curve is the parabola (equation (1.5)), predicted on the basis of tidal friction.

The hard limits on ΔT derived from untimed observations of total and annular solar eclipses are collected in the electronic supplementary material, table S10, and plotted in figures 11 and 12. Generally, one hard limit and the direction of the solution from large partial solar eclipses (apart from in 120 and 1004) are collected in the electronic supplementary material, table S11, and plotted in figures 13 and 14. The other limit—on the other side of totality—is usually uncritical or redundant when fixing ΔT. The uncritical values are shown in square brackets, and the redundant values are omitted in the electronic supplementary material, table S11. In 822, there were reports of totality in N. China; hence, the central zone must have passed north of the capital, and only an upper bound is viable. The lower limit is indicated by dashes. Similarly, in 1605, the southern portion of the Sun was said to be obscured; hence, only the upper bound is viable. For the medieval eclipses 1133, 1147 and 1178, there are contemporaneous observations of totality, which allow us to discard the lower bound. All of the hard ΔT limits prior to 1500 are quoted to the nearest 20^s^.

Other untimed results (electronic supplementary material, tables S12 and S13; all from Babylon), which produce a firm limit and direction of solution, have been added to figure 13 as follows: solar eclipses where the Sun rose or set eclipsed (three observations); lunar eclipses where the Moon rose or set eclipsed (seven observations). Discrete points from the estimates of the degree of obscuration of lunar eclipses at rising or setting are collected in the electronic supplementary material, table S14, and plotted in figure 13 (five observations).

The weighting system for the Babylonian observations, which is based on relation (3.1), is distinct from the weighting of observations from other later sources. As there is no overlap between the Babylonian and other timed observations, apart from a few Greek observations (figure 9), this disparity in the weighting schemes does not affect the spline fit.

## 4. Fitting smooth curves to ΔT

### (a) Parabolic fit, −720 to 2015

The following parabola is a good fit overall to the values of ΔT discussed in §3 and plotted in figures 9, 11–14 (it lies outside the box in figure 10).

4.1 $$\Delta T=-320.0+(32.5\pm 0.6){\left(\frac{\mathrm{year}-1825}{100}\right)}^{2}\;s.$$

**Figure 15.**
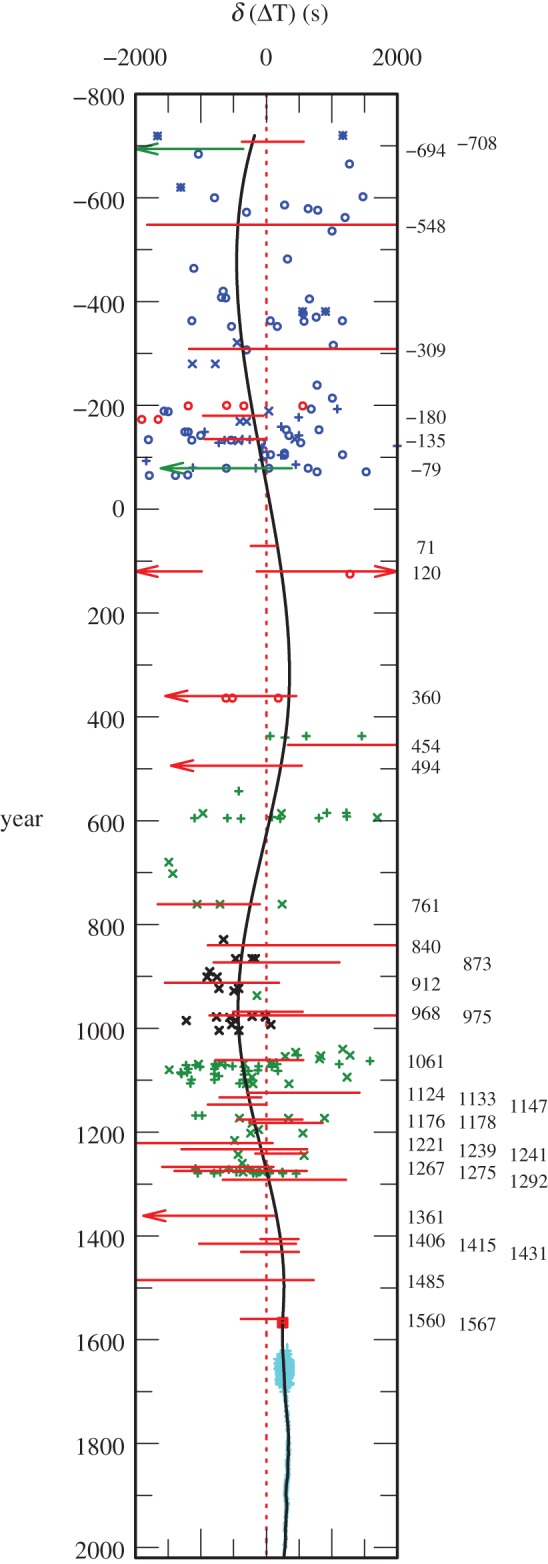
Plot of the residuals *δΔT* with respect to the parabola (4.1) represented as a straight line. The key to the symbols is as in figures 9–14. The black curve is the spline fit discussed in §4b.

It was derived iteratively by subtracting preliminary solutions for the parabola from the timed and untimed results for ΔT. Figure 15 shows the residuals of ΔT with respect to this adopted parabola. Only the critical observations in figures 11–14 are included in this figure. The epoch (1825) of the apex of the parabola (equation (4.1)) is somewhat arbitrary, and is ultimately dependent on the mean epoch of the solar observations used by Newcomb in deriving the constants of his *Tables of the Sun* [23]. He determined the mean motion of the Sun on the UT scale from observations of its position between 1750 and 1892. This value of the mean motion of the Sun was used in the definition of the unit of the second on the Ephemeris Time (ET) scale, which was carried through to the TT scale and the definition of the SI second. Thus, the apex of the parabola, at which the average length of the mean solar day is equal to 86400^s^ SI, falls somewhere near 1825. There is no other *a priori* constraint on the positioning of the parabola. The parabolic coefficient +32.5±0.6 in (4.1) is an improvement on the result +31.0±0.9 in our previous paper [1].

However, the parabola, shown as a dotted line at zero in figure 15, is not a good fit everywhere, particularly around 1000. Nearly all the Arab solar eclipse timings (black crosses) lie below the parabola, and all the timed (occultation) data after 1600 lie above the parabola. In addition, a careful examination of the untimed observations in figure 15 supports our conclusion that a parabola does not satisfy all the observations. The limits on ΔT imposed by the following eclipses stand off from the parabola: −694 (lunar), 454, 761, 1133, 1567.

In principle, a major source of error in the limits of total solar eclipses would arise either from a misidentification of the site of observation of the eclipse or from the possibility that the eclipse was partial rather than total. We have taken considerable care on both counts, and have only retained observations where the place and description of the eclipse are both definite. It must be emphasized that the timed and untimed data are not only independent of one another, but also both sets contain data from different civilizations. The commentaries on these critical eclipses and others in the following discussion are collected in the electronic supplementary material, section S3.

### (b) Fitting a curve by cubic splines

The accuracy and density of observations varies considerably over the period −720 to 2015. We chose to fit the data by cubic splines because of their flexibility in the placing of knots and the continuity of their first derivative, which corresponds to the change in the length of the mean solar day (lod). This geophysical parameter is useful in applying a control on the magnitude and temporal behaviour of the fluctuations that might realistically be expected in ΔT, judging by its behaviour after 1600. The actual value of ΔT at epoch 1900 should be close to zero, as distinct from the value on the parabola in §4a, which follows the long-term trend over centuries. This is a consequence of the historical definition of ΔT (*Explanatory Supplement* [20, section 2.551]), and is somewhat arbitrary. The precise value of ΔT is dependent on the lunar ephemeris used in the reduction of the observations.

Cubic splines were fitted to the weighted values of ΔT (see §3b) with knots spaced at intervals to reflect the accuracy and density of the observations: −720, +400, +1000, +1500, +1600,+1650,+1720. Starting with +1800 the knots were spaced as follows

$$\begin{array}{cc} 1800.0-1900.0; & 5\text{ year intervals}\\ 1900.0-2016.0; & 3\text{ year intervals}\text{.} \end{array}$$

We used an iterative procedure to arrive at this optimum separation of the knots for the various datasets. In the period 1962–2015, where TAI−UT1 is available, this involved trial solutions with separations ranging from 2 to 5 years. The first derivative along the fitted curves gives the lod, which is compared with the IERS values [19].

**Figure 16.**
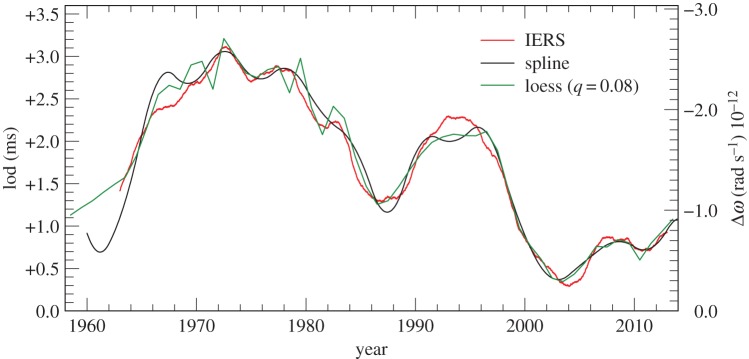
lod 1960–2015 derived from lunar occultations: black curve from cubic splines with knots at 3 year intervals; green curve from fitting by loess with smoothing parameter *q*=0.08. Lod 1962–2015 from IERS data smoothed by 2 year moving average. (The conversion factor for deriving the change in the rotational velocity Δ*ω* in rad s^−1^ from the lod in ms is −0.843×10^−12^ lod.)

**Figure 17.**
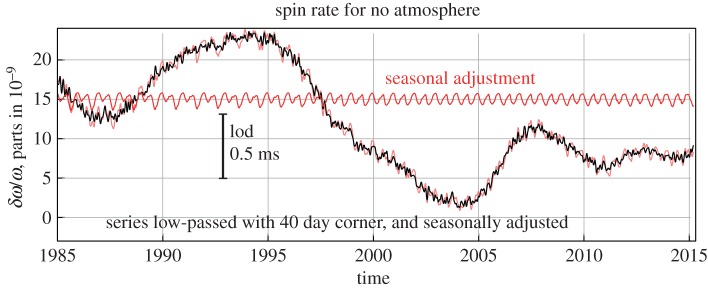
lod 1985–2015 with atmosphere angular momentum subtracted.

The IERS lod data are tabulated at daily intervals and they display the annual fluctuations in the Earth’s rotation owing to angular momentum transfer between the atmosphere and the mantle, as well as longer-term fluctuations. We have smoothed the IERS data by taking a 2 year moving average. This is plotted in figure 16 together with the occultation results for lod. This degree of smoothing is supported in the years 1985–2015 by the behaviour of the lod with the atmospheric angular momentum subtracted (figure 17). A 3 year interval for the knots in the spline fitted to ΔT produced the best agreement between the lod from the occultations and the IERS data. This gives us a measure of confidence in the resolution we might expect from the occultation data prior to 1962.

Before 1600, the timed observations alone were not sufficient to fix the behaviour of ΔT, particularly in the interval −100 to 800 where the timed observations are sparse and relatively inaccurate (figure 15). Critical limits from the following untimed observations of eclipses were used to supplement the timed data: −708,−694,−135,71,454,761,1241,1567. These eclipses are crucial in defining the fit of the spline before 1600, and their selection for this purpose requires some justification. To this end, commentaries on these eclipses are included in the electronic supplementary material, section S3. The critical limits from these selected untimed observations were assigned high weight and combined with the weighted timed observations to arrive at a final curve fitted by cubic splines, with knots spaced as before.

The lower limit of the total solar eclipse (Chinese) of −708 in figure 11 and the upper limit of the lunar eclipse (Babylonian) of −694 in figures 13 and 15, where the eclipsed Moon set before sunrise, are critical in constraining the value of ΔT close to +20 000^s^ around the epoch −700. Both eclipse reports are very dependable, and these form a tight constraint on the solution during a period when the timed Babylonian data are less accurate because the times were expressed to the nearest 5^°^, or even 10^°^, equivalent to 1200^s^ and 2400^s^.

The lower bound of 454 and the upper bounds of 71 and 761 constrain the spline curve at a time that is almost devoid of accurate timed observations. Likewise, 1361 and 1567 set tight constraints for the period 1300–1600. The reliability of these eclipses is discussed in the commentaries in the electronic supplementary material, section S3.

The resultant spline curve is plotted in figures 9–14. In figure 15, it is plotted with respect to the parabola (4.1).

The goodness-of-fit of this curve before 1600 is tested against the limits imposed by all the untimed data listed in the electronic supplementary material, tables S10–S13. From figures 11 and 12, it will be seen that the spline satisfies all the constraints of the total and annular solar eclipses, except that of −600. Of course, this is necessarily the case for the few eclipses listed above which were used in fitting the spline. The large partial solar eclipses and rising and setting lunar eclipses in figure 13, and the large partial solar eclipses in figure 14, also satisfy the spline curve, except for that of −382. These two exceptions (−600 and −382) require further investigation (see electronic supplementary material, section S4). Regarding the commentary for −600 in the electronic supplementary material, section S4, it will be seen that this report does not have confirmatory evidence from the Hanshu, as distinct from −708, which does. To accommodate the result from −600, along with those of −708 and −694, requires a large variation in the lod, which is geophysically implausible. This is also the case with the −382 eclipse in the *Almagest*. It is one of a group of observations discussed by Ptolemy. There is always a slight risk in using the Babylonian data from the *Almagest* because they reached Ptolemy through the intermediary of Hipparchus, who lived more than three centuries before Ptolemy.

Another discrepant untimed total solar eclipse which we have rejected is that of 1605. This was reported to have been total at Marseilles, but this is unlikely to have been the case (see electronic supplementary material, section S4). For this reason, we have not listed it in the electronic supplementary material, table S10, nor plotted it in figures 12 and 15. On the other hand, the observation reported nearby of a large partial solar eclipse is plotted in figure 14.

### (c) Fitting a smooth curve after 1600 using local polynomial regression

Figure 10 shows fluctuations on a time scale of decades and it is interesting to investigate their spectral characteristics. To do this, we prepared a dataset of lod values, which has less smoothing than the spline. There are sufficient values of ΔT after 1600 to fit a smooth curve using local polynomial regression, otherwise known as loess. We have used a version of the method by Cleveland *et al.* [24], with a quadratic smoothing parameter throughout, and the fraction (*q*) of the points used in computing each fitted value as follows

$$\begin{array}{ccccccc} \text{span} & 1550-1750 & 1620-1750 & 1700-1810 & 1800-1900 & 1890-1955 & 1940-2015\\ q & 1.0 & 0.75 & 0.33 & 0.33 & 0.1 & 0.08 \end{array}$$

These values were chosen in accordance with the density of the data points and in an iterative process using the behaviour of the first derivative (lod) as a control, as with the spline fit. The overlap in the partitioning of the dataset enabled a smooth transition across the whole span of time.

## 5. Change in the length of the day

### (a) Variations in lod

**Figure 18.**
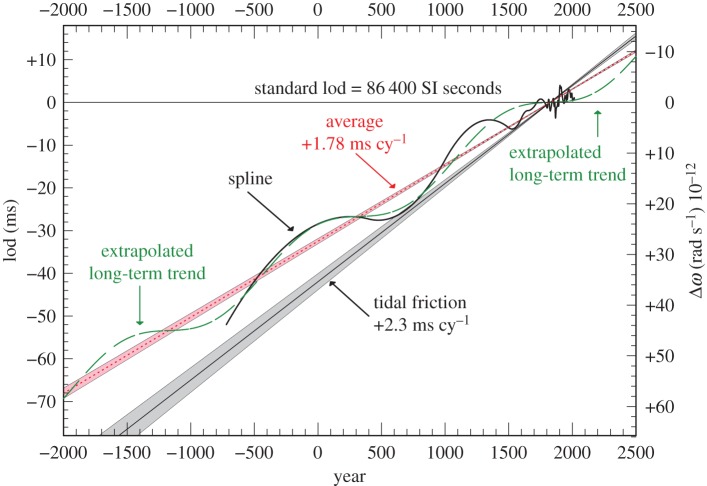
lod −2000 to 2500. The dotted red line is the average measured rate of change in the lod, +1.78±0.03 ms cy^−1^, which is equivalent to an acceleration of −4.7±0.1×10^−22^ rad s^−2^. The shaded grey area shows the change expected on the basis of tidal friction, +2.3±0.1 ms cy^−1^, equivalent to −6.2±0.4× 10^−22^ rad s^−2^. The black curve is the slope on the spline fit shown in figures 9 and 10. The green-dashed curve is the extrapolation of the oscillation (equation (5.1)).

The first time derivative along the ΔT spline curve in figures 9, 10, and in figure 15 on an expanded scale with respect to the parabola (4.1), gives the change in the rate of rotation of the Earth, which can be expressed as the lod in milliseconds from the adopted standard of 86400^s^ SI. This is plotted in figure 18. The long-term average rate of change in lod derived from the time derivative of equation (4.1) is +1.78±0.03 ms cy^−1^, where the time in centuries is reckoned from the epoch of the apex of the parabola, 1825. This result compares with that of +1.70±0.05 ms cy^−1^ in Stephenson & Morrison [1]. On the basis of tidal friction, the calculated change is +2.3±0.1 ms cy^−1^.

**Figure 19.**
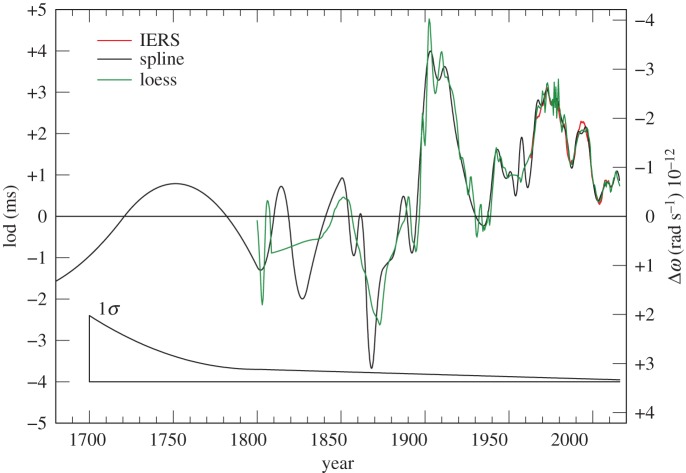
lod 1700–2015 derived from lunar occultations. The black curve is the slope on the spline curve displayed in figure 10. The green curve is the half-yearly values derived from the loess smoothing after 1800 as described in §5b. The red curve is the IERS data as displayed in figure 16.

After 1600, the higher resolution afforded by telescopic observations reveals the decade fluctuations which are usually attributed to core–mantle coupling [25–28]. These are plotted in figure 19 on a larger scale. Similar fluctuations are undoubtedly present throughout the whole historical record, but the low accuracy of the data is not capable of resolving them. All that can be resolved is a long-term oscillation with a period of about 1500 years and amplitude comparable to the decade fluctuations. In figure 18, it is plotted from the expression

5.1 $$\mathrm{lod}=+1.78\;t-4.0\sin2\pi \left(\frac{t}{15}\right)\;\mathrm{ms},$$

where *t* is measured in centuries from 1825.

The reality of the quasi-periodic behaviour of the lod should be treated guardedly. It may simply represent the envelope of stochastic processes, rather than evidence of a periodic change. The periodic model does not fit well with the lod around 1500, where its behaviour is dictated by the requirement that the spline satisfies the total solar eclipses of 1361 and 1567 (see electronic supplementary material, section S3), as shown in figures 14 and 12, respectively, and also in figure 15. The apparent period of 1500 years is driven to some extent by the spacing of the knots in the spline fit described in §4b. The number and placing of the knots was chosen such that the resultant amplitudes of the fluctuations in the lod displayed in figure 18 were kept to a minimum, conversant with the requirement of satisfying the values of *δ*(ΔT) displayed in figure 15. The introduction of more knots to try to satisfy the rejected observations discussed in the electronic supplementary material, section S4, leads to fluctuations in the lod with considerably greater amplitudes. We considered these to be unrealistic.

It is evident from figure 15 that there are fluctuations in ΔT on a time scale greater than decades. The decade fluctuations are barely discernible on the scale of figure 15. From figure 15, it can be seen that were it not for the upper bound of the untimed eclipse −694 and the lower of 454, the dotted straight line representing the parabola (4.1) would be a satisfactory fit to all the data before 700. We have already discussed the reliability of these critical eclipses in §4b, and we have no reason to mistrust these any more than the others. After 700, it is clear that one parabola does not fit the observations on a time scale of centuries. If the fluctuations before 700 are spurious, then a mechanism is required to cause fluctuations on a time scale of centuries after 700, but not before.

The extrapolation of the lod beyond the limits of the dataset is dependent on the reality of the 1500 year oscillation. It is also assumed that tidal friction is essentially unchanged to within the boundaries of its uncertainty in the period −2000 to 2500. Both are somewhat conjectural and require further investigation in the future.

### (b) Spectral analysis of lod after 1800

After 1800, there appears to be sufficient detail in the lod shown in figure 19 to make an estimate of the power spectrum density (PSD) worthwhile. To do this, we employed an adaptive sine multitaper method made available by Barbour & Parker [29]. This method is efficacious for data of large dynamic range with narrow and wide-band features.

The occultation data 1800–2015 enabled us to apply this PSD analysis to a span of 215 years. The values of ΔT were evaluated at half-yearly intervals in the loess smoothing of §4c and the lod obtained by applying a quadratic convolute with 11, 11, 7, 5, 5 points up to 1955, and first differences in the last period, 1940–2015. These are plotted in figure 19.

**Figure 20.**
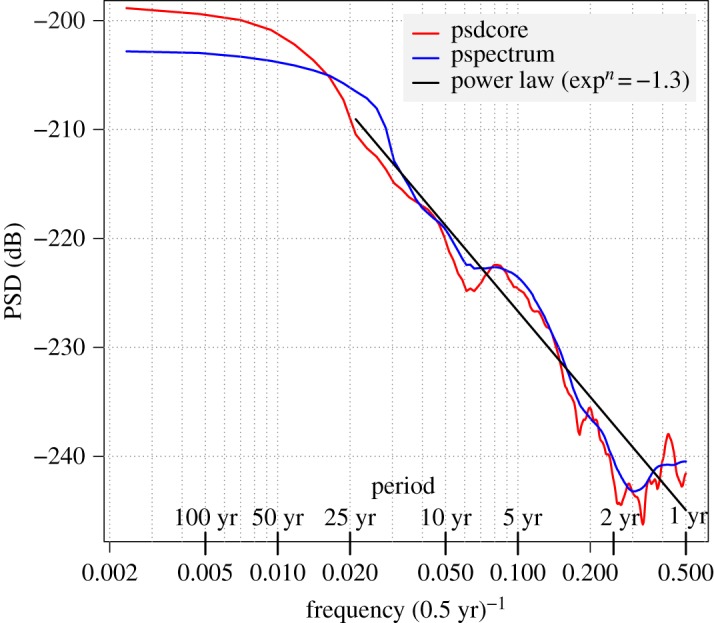
Power spectrum density (PSD) of half-yearly lod values in the years 1800–2015 obtained from the slope on local polynomial regression of occultation ΔT values.

These lod data are independent of the IERS dataset which spans 1962–2015. The resultant spectrum is plotted in figure 20. This shows a power law with exponent −1.3 operating in the range 2–30 years. The spectrum also shows an increase in power at a period of about 6 years, which supports conclusions by Abarca del Rio *et al.* [30], Gillet *et al.* [31], Holme & de Viron [32] and Gillet *et al.* [28], based on earlier lod series incorporating IERS data.

## 6. Long-term accelerations in rotation

The best-fitting parabola to the observations (red dotted curve in figures 9, 11–14) corresponds physically to an acceleration of −4.7±0.1×10^−22^ rad s^−2^ in the rotation of the Earth. This is significantly less algebraically than the acceleration expected on the basis of tidal friction alone (equation (1.3)), −6.2±0.4×10^−22^ rad s^−2^ (shown shaded in the figures). Some other mechanism, or mechanisms, must account for the accelerative component of +1.5±0.4×10^−22^ rad s^−2^. This non-tidal acceleration is probably in part associated with the rate of change in the Earth’s oblateness attributed to viscous rebound of the solid Earth from the decrease in load on the polar caps following the last deglaciation [33]. However, by itself, this mechanism cannot account completely for the non-tidal acceleration, and some additional correction for core–mantle coupling is required [34].

## 7. Conclusion

An exhaustive search of historical records of eclipses of the Sun and Moon extant from Babylonia, China, Greece, the Arab Dominions and Europe has produced the most comprehensive list of observations to date, which are germane to the study of the variability of the Earth’s rate of rotation since 720 BC. The eclipse observations are not only independent by provenance, but also by methodology. The eclipses are analysed in two main groups: timed and untimed. Taken independently and in combination, they produce reliable results for the Earth’s rotation.

Assuming that the measurement of tidal braking in the Earth–Moon–Sun system is secure, our main conclusion is that this mechanism alone does not account for the observed deceleration in rotation over the past 2700 years. A smaller accelerative component of +1.5±0.4×10^−22^ rad s^−2^ is also present, which is thought to arise from a combination of post-glacial rebound and core–mantle coupling.

Evidence is found for fluctuations in the rate of rotation on a time scale of centuries to millennia. These appear to have a quasi-periodic behaviour of about 1500 years with a comparable amplitude to the decade fluctuations. However, this is less reliably established because it is critically dependent on relatively few observations. Nevertheless, this oscillatory model provides the most satisfactory agreement overall with observation.

We are not aware of any other eclipse observations before about 720 BC that can contribute significantly to the measurement of the Earth’s rotation in the historical past.

After AD 1600, timings of lunar occultations of stars provide the most accurate measure of the Earth’s rotation on time scales longer than about a year, until the introduction of the atomic time scale from 1962 onwards. A recent compilation and reduction of all the occultation observations has enabled us to refine previous results for the decade fluctuations in the Earth’s rotation. The PSD of the occultation lod values in the years 1800–2015 reveals a power law with exponent −1.3 for periods in the range 2–30 years, and an indication of an increase in power around a period of 6 years, in agreement with investigations by other authors.

It is our aim that the data in this paper will set boundary conditions for future geophysical studies of core–mantle coupling, post-glacial isostasy and sea-level changes in the past 2700 years.

## Supplementary Material

The Supplement

## Supplementary Material

ASCII Data Text Files

## Data accessibility

Web pages are available from H.M. Nautical Almanac Office’s website at http://astro.ukho.gov.uk/nao/lvm, which contain tables of lod and ΔT at various intervals, together with estimates of their errors and related material.
