# Supplementary material for: Measurement of the Earth's rotation: 720 BC to AD 2015
Source: Proc Math Phys Eng Sci. 2016 Dec;472(2196):20160404. doi: 10.1098/rspa.2016.0404 (PMC5247521; doi:10.1098/rspa.2016.0404)
Supplement: The Supplement [file rspa20160404supp1.pdf]

PROCEEDINGS A

rspa.royalsocietypublishing.org

Research

Article Published 2016 December

Subject Areas:
observational astronomy, Solar System, stars

Keywords:
eclipses, occultations, length of day, tidal friction, core-mantle coupling, sea-level

Author for correspondence:
L. V. Morrison
e-mail: LMorr49062@aol.com

Published Paper
http://dx.doi.org/10.1098/rspa.2016.0404

Measurement of the Earth's rotation: 720 BC to AD 2015: The Supplement

F. R. Stephenson[1], L. V. Morrison[2] and C. Y. Hohenkerk[3]

[1]University of Durham, Durham, UK
[2]Pevensey, East Sussex, UK
[3]HM Nautical Almanac Office, UK Hydrographic Office, Taunton, TA1 2DN, UK

New compilations of records of ancient and medieval eclipses in the period 720 BC to AD 1600, and of lunar occultations of stars in AD 1600–2015 are analysed to investigate variations in the Earth's rate of rotation. It is found that the rate of rotation departs from uniformity, such that the change in the length of the mean solar day (lod) increases at an average rate of $+1.8$ ms per century. This is significantly less than the rate predicted on the basis of tidal friction, which is $+2.3$ ms per century. Besides this linear change in the lod, there are fluctuations about this trend on timescales of decades to centuries. A power spectral density analysis of fluctuations in the range 2-30 years follows a power law with exponent -1.3, and there is evidence of increased power at a period of 6 years. There is some indication of an oscillation in the lod with a period of roughly 1500 years. Our measurements of the Earth's rotation for the period 720 BC to AD 2015 set firm boundaries for future work on post-glacial rebound and core-mantle coupling which are invoked to explain the departures from tidal friction.

S1. Introduction

The main paper, this supplementary material and the ASCII files of all the tables are available for download from the Royal Society Proceedings A website (see link above).

The electronic supplementary material is available online at rs.figshare.com.

© The Authors. Published by the Royal Society under the terms of the Creative Commons Attribution License http://creativecommons.org/licenses/by/4.0/, which permits unrestricted use, provided the original author and source are credited.

THE ROYAL SOCIETY
PUBLISHING

This supplement contains five sections of supplementary material comprising:

S2. Comparative accuracy of Babylonian measurements of time intervals
S3. Commentary on eclipses which are critical in defining the spline fit to ΔT
S4. List of rejected eclipses before 1600
S5. Polynomial coefficients (see table **S15**) for evaluating the spline fit to ΔT
S6. Tables (**S1**-**S14**) of observational values of ΔT before 1600.

All the tables are available in ASCII text files named `Table-Sxy.txt` where `xy` is the table number. They are collected together, with the extracted data from the lunar archive of occultations (Herald & Gault [3]) into the file rspa.2016.0404.tables.txt.zip, which is available from the data tab of this website.

S2. Comparative accuracy of Babylonian measurements of time intervals

Figure S1. Babylonian values of ΔT for observations with negative time intervals. The observations before −560 are intrinsically less accurate (indicated by a vertical dotted line).

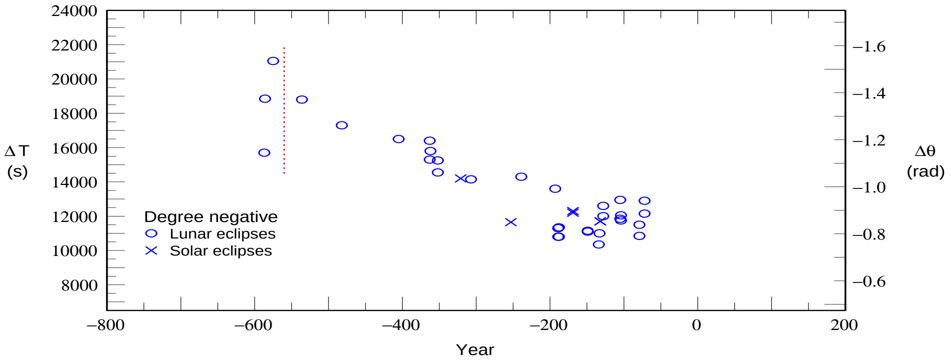

Figure S2. Babylonian values of ΔT for observations with positive time intervals.The observations before −560 are intrinsically less accurate (indicated by a vertical dotted line).

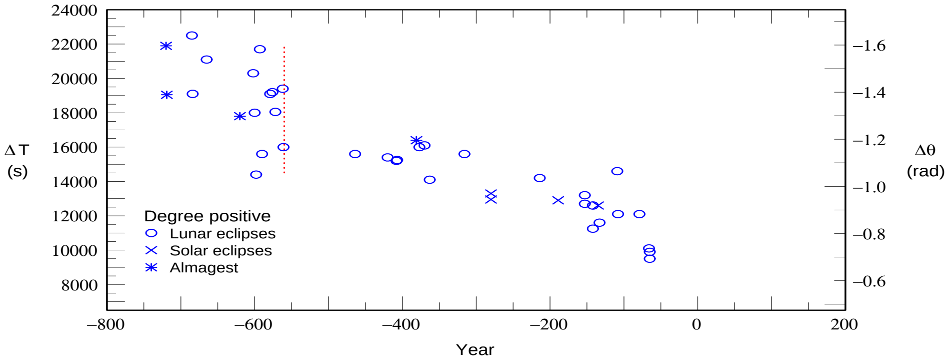

We investigated the comparative accuracy of lunar eclipses timed with respect to sunset (denoted by + degrees in table **S1**) and timed before sunrise (− degrees). The equivalent procedure for solar observations is timings either after sunrise or before sunset (table **S3**). Both sunrise and sunset would normally be well-defined moments. The positive and negative degree timings, which are not distinguished in figure 2 of the main paper, are plotted separately in figures **S1** and **S2**. The scatter is similar—discounting the early imprecise observations before −560. This is slightly surprising, because the negative degree timings could sometimes cover a considerable

elapsed time between the first timing to the last timing just before sunrise (for a lunar eclipse) or near sunset (for a solar eclipse). Two such cases are −189 and −128, where the last timing is given high weight because the number of degrees before sunrise is small (10° in −189, and 15° in −128), even though the elapsed time after first contact is considerable (40° in −189, and 55° in −128). Of course, much depends on the methodology of the timing, presumably using a clepsydra. For instance, if a clepsydra was started at the first phase of the eclipse, and then 'read off' for other phases until sunrise, then these results imply that the clepsydra may have been rated again at sunrise and the timings adjusted retrospectively.

rspa.royalsocietypublishing.org Proc R Soc A 2016.0404 (S)

S3. Commentary on critical eclipses

−708 Jul 17 (China)

This record is one of more than 30 brief accounts of solar eclipses in the *Chunqiu* (*Spring and Autumn Annals*), a chronicle of the small Chinese state of Lu covering the interval from −721 to −480, and compiled soon after this era. Two other eclipses, occurring in −600 and −548, are also described as total in the *Chunqiu* (see below). The report of the eclipse of −708 in the *Chunqiu* may be translated as follows: "The Sun was eclipsed; it was total (*ji*)." This is the earliest direct record of a total solar eclipse in the history of any civilisation. In the *Hanshu* (Ch.27)—a much later source—we find the following record relating to the same eclipse: "The Sun was eclipsed; it was total . . . . The solar eclipse penetrated centrally through it (the Sun); above and below it were remarkably yellow." This seems to suggest a total eclipse with a bright corona. For totality at Qifu, $20160^s < \Delta T < 21100^s$. This was a narrow zone of totality, moving at a large angle to the equator. Hence the computed magnitude is very sensitive to small changes in ΔT. The Lu capital was Qifu (35.53°N; 117.02°E). As for other eclipse records in the *Chunqiu*, the year is expressed in terms of the reign of the appropriate Duke of Lu, while the lunar month and day of the 60 day cycle are also given.

Computations reveal that with only a very few exceptions, all the recorded dates of solar eclipses in the *Chunqiu* are exactly correct. This suggests a high degree of organisation at this early period, which presumably would be only possible if the records are from the Lu capital itself. Confirmatory evidence is provided by the reports of three of the eclipses, occurring in −668, −663 and −611. In each case eclipse ceremonies held at Qifu are described in the *Chunqiu*, although none of these three eclipses was said to be total.

−694 Apr 30/May 1 (Babylon)

The computed magnitude of this lunar eclipse is 0.74 at Babylon (32.55°N; 44.42°E). After giving the date, the slightly damaged record states "[. . .] total. It set eclipsed. Began at $30 + [x]$ deg before sunrise" (trans. Hunger [5, vol. 5 p. 5]). According to Huber and DeMeis [4, p. 4]: the text may be translated as: "not complete, it set eclipsed. (Began) at 30° before sunrise." The only useful detail is that the Moon set whilst eclipsed. Assuming an observer at an elevation of 10 to 15 m above the ground (the height of the walls of Babylon), and horizontal refraction as 34′, the true lunar altitude, corrected for parallax, would need to be $-0\overset{\circ}{.}4$ for the whole Moon to be visible. The time of moonset is also a function of ΔT and a certain amount of iteration is needed. Hence for last contact after moonset (Moon to set eclipsed), $\Delta T < 19950^s$.

−180 Mar 4 (China)

This was described as "total" in the *Hanshu* (Ch.27), while a separate history—the *Shiji* (chapter 9)—states that "it became dark in the daytime". In both texts, it is asserted that unpopular Empress Dowager was disturbed, saying "This is on my account". There is nothing in either text to suggest observation outside the capital of Chang'an, and the direct allusion to the Empress Dowager would seem to confirm this. The eclipse would be total at Chang'an (34.35°N; 108.88°E) for values $11780^s < \Delta T < 12700^s$.

−135 Apr 15 (Babylon)

Among the many solar eclipse reports from Babylon, only in this single instance (in −135) is there clear evidence of totality at Babylon. Two accounts are preserved on separate texts: a "goal year text" containing observations which were assembled to assist predictions of future events; and an astronomical diary. The records may be translated as follows:

(i) "In 18° of daytime it made a total (eclipse). (It began) at 24° after sunrise." (trans. Hunger [6, p. 269]).

(ii) "At 24° after sunrise... [Ven]us, Mercury and the Normal stars were visible; Jupiter and Mars, which were in their period of visibility (i.e. they had set heliacally) were visible in its eclipse ... 35° onset, maximal phase and clearing." (trans. Sachs and Hunger [7, p. 185]).

Here we have perhaps the finest accounts of a total solar eclipse from antiquity. For totality at Babylon, $11220^s < \Delta T < 12140^s$. The timings of the various stages of this eclipse also lead to individual results for ΔT.

71 Mar 20 (Greece)

Plutarch (trans. Chermiss and Helmbold [1]) described a recent eclipse of the Sun in the following words: "If you will call to mind this conjunction which, beginning just after noonday, made many stars shine out from many parts of the sky and tempered the air in the manner of twilight." Later in his dialogue, Plutarch remarked: "Even if the Moon does sometimes cover the Sun entirely, the eclipse does not have duration or extension; but a kind of light is visible about the rim which keeps the shadow from being profound and absolute." Here Plutarch seems to be describing the solar corona. No other ancient or medieval writer mentions the corona until as late as AD 968 (as seen in Constantinople). Although this latter description has been suggested as an annular eclipse, no annular eclipse was visible in or near Greece during the lifetime of Plutarch.

Plutarch was born at Chaeroneia around AD 46 and died after AD 119. He was normally resident in Chaeroneia throughout his life, but he is known to have travelled throughout central Greece and to Rome on official visits. He also had close links with the Athenian Academy. The beginning of his dialogue De Facie is lost. However, his many dialogues are usually set in various places in central Greece, including Sparta, but sometimes in Rome—the places with which he himself was familiar.

Between AD 50 and AD 119, only four eclipses were large in Greece or Rome: AD 59 (total), 71 (annular-total); 75 (annular-total) and 83 (total). At this period, ΔT was around 9600^s. It seems likely that the eclipse would be unexpected and hence only noticed shortly before totality. [Place coordinates: Athens: 37.98°N, 23.73°E; Chaeroneia: 38.40°N, 22.90°E; Rome: 41.90°N, 12.50°E.]

(i) AD 59 Apr 30: Plutarch was then only aged 15 and whenever he wrote his dialogue the eclipse could thus scarcely have been described as "recent". Further, the track of totality ran almost along a line of latitude in the Mediterranean, and the eclipse could never have been total further north than lat 36°.3. Rejected.

(ii) AD 83 Dec 27: This passed far to the east of Greece and Rome, and could never have been total there. Rejected.

(iii) AD 75 Jan 3: For totality on mainland Greece, the value of ΔT would have had to have been at least 1500^s less than the value of 9600^s, based on the parabola (4.1). Also, the eclipse would have occurred well into the afternoon (between $15^h.7$ and $15^h.8$ LT), by which time the Sun's altitude would have been between 7°.0 and 7°.5. It could scarcely be described as beginning just after noonday. Rejected.

(iv) AD 71 Mar 20: For a value of ΔT in the range 9440^s to 9830^s, this eclipse would have been total on mainland Greece somewhere between Chaeroneia and Athens. Thus it could

have been witnessed by Plutarch, and we adopt this as the only total eclipse to satisfy the record. For a value in excess of 9830^s the eclipse would not have been total anywhere on mainland Greece, and we regard this as an upper bound. The computed local time of the eclipse is around 11 a.m., which is in adequate accord with the informal statement of 'just after noonday', as the eclipse would only have been noticed when it approached totality.

454 Aug 10 (China)

This eclipse is described in the following words in the "Five Phases" Treatise (chapter 34) of the *Songshu* (*History of the (Liu) Song Dynasty*). "The Sun was eclipsed; it was total. The constellations were very bright." Here the report of totality is affirmed by the allusion to the brightness of the stars. At the time, the capital was located at Jiankang (Nanjing; 32.03°N; 118.78°E). Although the place of observation is not formally stated in the text, the capital—where the imperial observatory (the source of a wide variety of observations of celestial phenomena) was located—seems highly likely. Only in one eclipse record in the entire treatise of the *Songshu* is the place of observation directly mentioned: in AD 429, when it is asserted that "the eclipse was not complete and like a hook. . . (but) in Hebei province (in northern China) the Earth was in darkness." In this latter case it may presumed that although only a large partial eclipse was visible at Jiankang, the occurrence of a more spectacular eclipse had been reported from the provinces. Only values of ΔT between 6120^s and 7900^s would lead to totality at Jiankang in AD 454. The lower ΔT limit is critical.

761 Aug 5 (China)

On this date we find a careful description of a total solar eclipse in the astronomical treatise (chapter 36) of the *Jiu Tangshu* (*Old History of the Tang Dynasty*). This may be translated as follows: "The Sun was eclipsed; the large stars were all seen. The Astronomer Royal, Chu Dan, reported: 'The Sun diminished. Precisely after 6 marks in the hour of *chen*, the loss began. Precisely after 1 mark in the hour of *si* it was total. At 1 mark before the hour of *wu* it was restored to fullness'." Briefer accounts confirming totality and the visibility of "all the large stars" are quoted in both the imperial annals (chapter 10 of the *Jiu Tangshu* and also in the astronomical treatise (chapter 32) of the *Xin Tangshu* (*New History of the Tang Dynasty*). Here we have one of the most detailed accounts of a total solar eclipse in Chinese history. The Tang capital, where the imperial observatory was situated, lay slightly to the south of the Han capital of the same name: (34.27°N; 108.90°E), and there can be no reasonable doubt that Chang'an was the place of observation in AD 761. The report of totality leads to values of ΔT between 1700^s and 3260^s, the upper limit being critical.

1124 Aug 11 (Europe)

Several chronicles of the Russian capital of Novgorod (58.50°N; 31.33°E) report a total eclipse of the Sun. The following example is clearly based on that of an eyewitness report (trans. Vyssotsky [12, p8]): "Before the evening service, the Sun began to diminish and perished completely. Great fright and darkness were everywhere. And the stars appeared and the Moon (sic). And the Sun began to augment and became full again and everyone in the town was very glad." (*Novgorodskaya I Letopic*). The reference to the appearance of the Moon here is obscure. However, the fact that the Sun "perished completely" leaves no doubt that this eclipse was total, as seen from Novgorod—the "town" as mentioned in the text. For totality at Novgorod, values of ΔT would need to be between 980^s and 2700^s. In particular, the lower limit of 980^s is fairly critical.

1133 Aug 2 (Europe)

Totality was observed at several places in Europe. A lower limit to ΔT is set by an observation at Reichersberg (48.34°N; 13.37°E), and an upper limit by an observation at Augsburg (48.37°N; 10.90°E). At Reichersberg, the annalist Magnus, reported that "Very many stars were seen. . . The Sun, as if it did not exist, was entirely concealed." (*Chronicon Magni Presbyterii*). At Augsburg,

Honorius related that "The whole sky was dark like night and stars were seen over almost the whole sky. Eventually, the Sun, emerging from the darkness, appeared like a star, afterwards in the form of a new Moon..." (*Summa Totius et Imagine Mundi*). For totality at Reichersberg, $520^s < \Delta T < 1740^s$, while for totality at Augsburg $-60^s < DT < 1160^s$. Combining these results, $520^s < \Delta T < 1160^s$ in 1133.

1241 Oct 6 (Europe)

This solar eclipse was independently observed to be total at two places in Europe: Reichersberg (48.34°N; 13.37°E) and Stade (53.60°N; 9.48°E). The eclipse records may be translated as follows:

Reichersberg: "The Sun, whilst it was bright, was suddenly covered with wonderful blackness a little after midday. As a result, no part of it could be seen and stars were seen as if at night" (*Chronicon Magni Presbyteri Continuatio*).

Stade: "There was an eclipse of the Sun ... some time after midday. Stars appeared and the Sun was completely hidden from our sight ..." (*Annales Stadenses*).

For totality at Reichersberg, $460^s < \Delta T < 1380^s$. At Stade, the corresponding limits are $620^s < \Delta T < 1630^s$. Combining these results, it may be concluded that in AD 1241, $620^s < \Delta T < 1380^s$. This is a fairly wide range but the lower limit is critical.

1361 May 5 (China)

Xu Zhentao, Pankenier and Jiang Yaotiao [13, p. 48] give translations of two very similar records of this eclipse—as seen at Songjiang (31.00°N; 121.22°E).

(i) "Emperor Shun of Yuan (dynasty), 21st year of the Zhizheng reign period, fourth month, day *xinsi*, the first day of the month. As the Sun was about to set, suddenly it was without brightness. It assumed the shape of a plantain leaf. The sky was dark as night and the stars and the Dipper shone brightly. In the time it takes to chew and swallow, the sky became bright again. Then in another short time the Sun set." (*Songjiang Fuzhi*). The *Songjiang Fuzhi* is a history of the town of Songjiang and was compiled by local government officials.

(ii) "(Same date). When the Sun was about 3 to 4 rods from setting, suddenly it was without brightness. Gradually it assumed the shape of a plantain leaf. The sky was briefly dark as night and the stars and the Dipper shone brightly. In the time it takes to chew and swallow, it was as before and the sky was bright again. The stars and the Dipper were also hidden. Then in another short time the Sun set." (*Chuogenglu*). The *Chuogenglu* is a privately compiled history of the Songjiang area, which was compiled in AD 1366. The Xu Zhentao et al [13]. make no further comment about this observation or state which of these two very similar records is the more original.

This eclipse was clearly very large at Songjiang, but nowhere does either text state that the Sun completely disappeared. However, the reference to the sky being as dark as night and the stars shining brightly implies a major eclipse. The comparison with a plantain leaf, which has long curving leaves, seems highly unlikely to refer to the corona. For instance, in his detailed investigation of historical observations of eclipses from various parts of the world, Stephenson [9] noted many total solar eclipses but could only find a single definite mention of the corona before AD 1600: in AD 968. On this occasion the corona was described as "a certain narrow feeble glow like a narrow headband." Hence we interpret the reference to the plantain leaf in 1361 as to the appearance of the Sun itself, reduced to a very thin crescent. The statement that the greatest phase lasted "for the time it takes to chew and swallow" clearly implies an extremely short time (perhaps only a few seconds).

In order for the eclipse of 1361 to have been partial at Songjiang, either $\Delta T < 500^s$ or $> 1760^s$. Only for values of ΔT very close to 500^s would this eclipse have been very dark (mag $\gg 0.998$),

in accordance with the vivid description. On this basis, maximal phase would be reached when the Sun's altitude was 2$^\circ\!\!.$2 in the west—in good accord with the record. The spline lies very close to the limit $\Delta T = 500^s$, and satisfies this constraint.

Interestingly, a further total solar eclipse AD 1575 May 10 is also recorded in the *Songjiang Fuzhi*. Although totality is clearly expressed, by this date ΔT would be so small that the observation is valueless: for totality, $-2150^s < \Delta T < +1390^s$.

1567 Apr 9 (Europe)

The prominent sixteenth century Jesuit astronomer Christopher Clavius was fortunate to observe two central solar eclipses only seven years apart. These events are reported in a treatise (Clavius [2]). Firstly, on 1560 Aug 21, whilst he was a student at the University of Coimbra (40.22°N; 8.42°W) in Portugal, he witnessed an impressive total eclipse of the Sun in which the Moon "covered the whole Sun for a considerable length of time." As a result there was darkness and stars appeared. [N.B. For this eclipse, if $\Delta T = 220^s$, the duration would be 0^s; with $\Delta T = 210^s$, duration would be 17^s; with $\Delta T = 200^s$, duration would be 53^s. Only this latter duration might be described as "a considerable length of time"!. Hence it may be concluded that in 1567 ΔT was probably less than about 200^s.]

Subsequently, on 1567 Apr 9, when Clavius was teaching at the Collegio Romano in Rome (41.90°N; 12.48°E), he observed a spectacular eclipse which was neither annular nor total. Clavius' description of this latter event may be translated as follows: "Although the Moon was placed between my sight and the Sun, it did not obscure the whole Sun as previously but . . . a certain narrow circle was left on the Sun, surrounding the whole of the Moon on both sides." As seen from Rome, the apparent lunar diameter would exceed that of the Sun by a factor of only 1.003. Allowing for the lunar limb profile, we estimate that only values of ΔT between 145^s and 165^s would satisfy the observation. Stephenson et al. [10].

S4. Rejected eclipses before 1600

−666 Oct 14/15 (Babylon)

Hunger [5, p. 11] translates the tablet as: "It set eclipsed. (Began) at 20 + $[x]$ before sunrise." Huber and DeMeis [4, p. 81] give the same rendering. We reject this entry since the sign specifying the time interval is damaged.

−600 Sep 20 (China)

This is the second of three solar eclipses, which are described as total in the *Chunqiu*. The text incorrectly gives the month as the seventh month. However, the written characters for 7 and 10 are very similar. Reading 10 in place of 7 gives the correct date. The record in the *Chunqiu* reads: "The Sun was eclipsed and it was total". In the *Hanshu* (Ch.27) it is also reported as total, but this time only with a comment about the error in the date. This eclipse would require a very high value of ΔT for totality at Qifu. Could the record have perhaps originated from another state? At this period, China was divided into a number of semi-independent states. For totality at Qifu, $21120^s < \Delta T < 21900^s$. This was also a narrow zone of totality, moving at a large angle to the equator. Hence the computed magnitude is very sensitive to small changes in ΔT. Interestingly, the *Chunqiu* reports a further total eclipse on a date corresponding to −548 Jun 19: once again, the text simply reports: "the Sun was eclipsed; it was total". This is again reported in the *Hanshu* (Ch.27) as total, but without further comment. Totality at Qifu produces a wide range of values $16160^s < \Delta T < 21640^s$ and these results clearly embrace both the parabolic fit and the spline fit (see figure 11). In summary, all, three eclipses were said to be total (*ji*). What is special about the eclipse of −708 is that the *Hanshu*, a later history, compiled several centuries after the event gives additional details which seem to affirm totality. No such support is given for −600 or −548.

−382 Dec 22/23 (Babylon?)

Here we have a record which states that the Moon set eclipsed. Most of the Babylonian eclipse records which we have investigated are preserved on the original cuneiform texts. However, the record in −382 is one of several instances in which we have only a late Greek copy—in Ptolemy's *Almagest* which was written in the second century AD, several centuries after the eclipses were observed. Regrettably, most of the cuneiform records of the Babylonian eclipses which Ptolemy cites are lost. Toomer [11], in the introduction to his careful translation of the *Almagest*, asserts that the earliest extant manuscripts of the *Almagest* date from the ninth century AD. Hence over the preceding centuries there could be a real possibility of copying errors. When dealing with eclipse timings, a scribal error just gives a false value of ΔT which merges with the rest of the results from the timed data. However, uniquely in −382 the report in the *Almagest*, which Ptolemy obtained from a record obtained by Hipparchus in the second century BC, also asserts that the Moon was still eclipsed when it set. Only for values of $\Delta T > 15700^s$ would the eclipse begin before moonset at Babylon; otherwise the eclipse would be invisible.

Figure S3. Plot of the values of ΔT from the lunar eclipses in the *Almagest*. The solid curve (black) is the spline fit described in section 4(b) of the main paper. The dotted (red) line is the parabola (4.1) in the main paper, and the shaded (grey) curve is the behaviour expected on the basis of tidal friction.

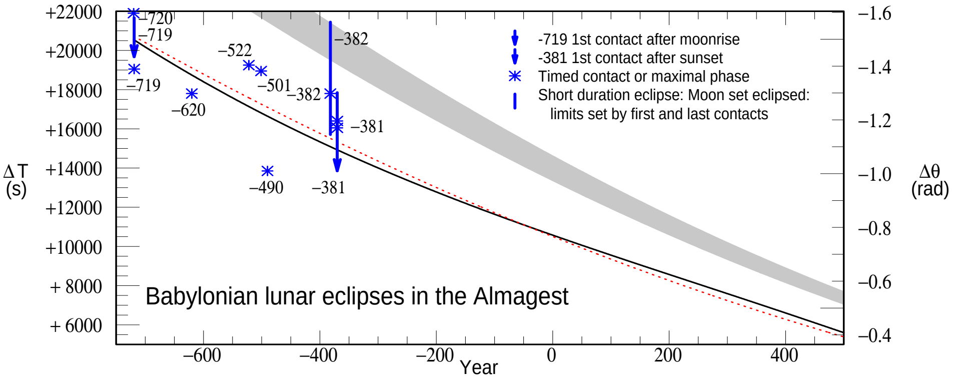

Such a high value of ΔT is in marked conflict with the results which we have derived from many other roughly contemporaneous observations. This leads us to question the reliability of the Ptolemaic record. It has been suggested to us by Christopher Walker (formerly of the British Museum) that the Greek text could be a description of the wrong eclipse. The date, expressed in the Greek style, is indeed equivalent to −382 Dec 23 on the Julian calendar. However, extant fragments of Babylonian eclipse tables group lunar eclipse records in a series of 19-year cycles. Hence—Walker suggests—a Greek scribe copying from a Babylonian tablet might have got the eclipse description in the wrong column. Whatever the explanation, we have no more than a fourth hand account—via a Babylonian scribe, Hipparchus, Ptolemy, and one or more medieval scribes—of the original record. We have thus decided to reject the preserved record.

The results for ΔT are listed in table **S4**, and included in figures 2 and 3 of the main paper, as well as separately here in figure **S3**.

−9 Jun 30 (Babylon)

This damaged text, which contains only 12 broken lines, is specifically devoted to a solar eclipse, which appears to have been total. The visibility of Venus, Mercury and *Sirius* during the eclipse, are mentioned. The date for this eclipse was derived using retrospective computation by J.M. Steele [8]. However, because neither the year nor the lunar month is preserved, we have therefore declined to use the observations (timed and untimed) in our investigation.

1605 Oct 12 (Marseilles)

This eclipse was reported as almost total at Forcalquier in Provence by the astronomer Wendelin (see table **S11** and figure 14 of the main paper. He gave a careful description of the appearance of the Sun "as a very narrow thread" and also remarked that he had heard that "in Marseilles the whole Sun appeared obscured with dense darkness". However, Wendelin does not name the observer, nor specify his source. Here we have an account at second hand. For totality at Marseilles, $+650^s < \Delta T < +1700^s$. The lower limit of $+650^s$ is incompatible with the highly reliable eclipse of 1567, which has an upper bound of $+165^s$, and also the timed occultation data (see figure 10 of the main paper). For these reasons we have rejected this observation.

S5. Polynomial coefficients for evaluating ΔT

The polynomial coefficients printed in table **S15** represent the spline approximation to ΔT (often written as Delta T) in seconds of time that is discussed in the main paper.

To evaluated ΔT for a given year and fraction (Y), find the row (i) from one of the 54 rows of the table **S15** such that

$$K_i \leq Y \leq^* K_{i+1} \tag{S5.1}$$

* Only use equality if $Y = 2016.0$. Then extract the interval (K_i, K_{i+1}) and the coefficients (a_0, a_1, a_2, a_3) from row i and calculate

$$t = (Y - K_i)/(K_{i+1} - K_i). \tag{S5.2}$$

Then

$$\Delta T = a_0 + a_1\, t + a_2\, t^2 + a_3\, t^3 \tag{S5.3}$$

where ΔT is in seconds and the coefficients and the interval are given in table **S15**.

Data Accessibility. Web pages are available from HM Nautical Almanac Office's website at http://astro.ukho.gov.uk/nao/lvm, which contain tables of lod and ΔT at various intervals, together with estimates of their errors and related material.

Authors' Contributions. F.R.S. delved into the eclipse archives and calculated the values of ΔT. L.V.M. carried out the analysis of the values of ΔT, and C.Y.H. obtained and ran the computer programs to produce the results and the figures.

Competing Interests. The authors declare that they have no competing interests.

Funding. No external funding was received.

Acknowledgements. We thank David Herald for providing details of his reduction of the occultation observations; Duncan Agnew for providing figure 17 in the main paper and suggesting the application of loess smoothing and power spectrum density analysis of the occultation data; Donald Starr for help in translating Chinese texts; Mathieu Dumberry, Richard Holme, Herman Hunger, Kurt Lambeck, John Steele and Arnold Wolfendale for helpful correspondence. The Fortran spline fitting routines are based on software made available by the National Institute of Standards and Technology. We also thank Steve Bell for help with graphics and the HMNAO website. We thank the United Kingdom Hydrographic Office for funding the processing charge for this Open Access article.

References

1. H. Cherniss and W. Helmbold. *Plutarch's Moralia*, volume XII. Heinemann, London, 1957.
2. C. Clavius. *In Sphaeram Ioannis de Sacrobosco, Commentarius*. Sumptibus Fratrum de Gabiano, Lugduni, 1593.
3. D. Herald and D. Gault. Lunar occultation archive, from 1623 to the present; updated 2015 October, Centre de Donnees Astronomique de Strasbourg, ftp download from http://cdsarc.u-strasbg.fr/viz-bin/Cat?VI/132B, 2012.

4. P. J. Huber and S. De Meis. *Babylonian Eclipse Observations from 750 BC to 1 BC*. Assoc. Culturale Mimesis, Milano, 2004.
5. H. Hunger. *Lunar and Planetary Texts in Astronomical Diaries and Related Texts from Babylonia*, volume V. Austrian Academy of Science, Vienna (Wien), Austria, 2001.
6. H. Hunger. *Goal Year Texts in Astronomical Diaries and Related Texts from Babylonia*, volume VI. Austrian Academy of Science, Vienna (Wien), Austria, 2006.
7. A. J. Sachs and H. Hunger. *Astronomical Diaries and Related Texts from Babylonia*, volume III. Osterreichische Akad. der Wissenshaften, Wien, 1996.
8. J. M. Steele. *The Latest Dated Astronomical Observation from Babylon*. In Archaeological Science 1997, A. R. Millard (ed.), Archaeopress, Oxford, 2001.
9. F. R. Stephenson. *Historical Eclipses and Earth's Rotation*. Cambridge University Press, 1997.
10. F. R. Stephenson, J. E. Jones, and L. V. Morrison. The solar eclipse by Clavius in A.D. 1567. *Astron. Astrophys.*, 322:347–351, 1997.
11. Toomer GJ (ed.). *Ptolemy's Almagest*. Springer-Verlag : New York, 1984.
12. A. N. Vyssotsky. Astronomical records in the Russian Chronicles from 1000 to 1600 AD. *Medd. fran. Lunds Astr. Obs. Historical Papers*, 22, 1949.
13. Xu, Zhentao, Jiang, Pankenier, and Yaotiao. *East Asian Archaeoastronomy: Historical Records of Astronomical Observations of China, Japan and Korea*. Gordon and Breach, Amsterdam, 2000.

The tables continue on the next page.

S6. Tables

Babylonian:	timed lunar eclipses (**S1**), lunar eclipses (culmination of ziqpu stars) (**S2**), solar eclipses (**S3**); Almagest timed and untimed lunar eclipses (**S4**); untimed solar eclipses (**S12**) and lunar eclipses (**S13**) rose or set eclipsed; lunar eclipses estimates of degree of obscuration at rising or setting (**S14**);
Chinese:	timed lunar eclipses (**S5**) and solar eclipses (**S6**);
Greek:	timed lunar and solar eclipses (**S7**);
Arab:	timed solar eclipses (**S8**) and lunar eclipses (**S9**);
Collected:	untimed total and annular solar eclipses (**S10**), partial solar eclipses (**S11**);
Spline fit:	polynomial coefficients for ΔT from −720.0 to 2016.0 (**S15**).

Table S1. Babylonian lunar eclipses

Year	ΔT(s)	Degree	Weight	Year	ΔT(s)	Degree	Weight	Year	ΔT(s)	Degree	Weight
−685	22500	+100	1	−377	16000	+37	3	−162	9650	−75	1
−684	19100	+20	6	−377	16200	+52	2	−162	9750	−65	1
−666	17550	+30?	0	−377	15750	+73	1	−159	14100	+48	2
−665	21100	+3	12	−377	14950	+92	1	−153	12700	+4	11
−602	20300	+50?	2	−370	12800	+66	1	−153	13200	+27	5
−600	18000	+95	1	−370	16100	+30	4	−153	13150	+48	2
−598	14400	+105	1	−370	15150	+52	2	−149	11100	−32	4
−593	21700	+10	10	−370	15500	+72	1	−149	11150	−12	10
−590	15600	+40	3	−370	14500	+92	1	−142	12600	+7	11
−587	15700	−20	6	−366	19600	−56	1	−142	11250	+30	4
−586	18850	−35	3	−363	14100	+10	10	−134	10700	−65	1
−579	19100	+45	2	−363	16400	−40	3	−134	7500	−35	0
−576	19200	+105	1	−363	15300	−14	8	−134	10350	−5	11
−575	21050	−40	3	−362	15800	−41	3	−133	11000	−9	10
−572	18050	+90	1	−362	15500	+64	1	−133	11600	+32	4
−562	19400	+6	11	−352	15950	−47	2	−128	11900	−55	1
−561	16000	+90	1	−352	14550	−24	5	−128	12000	−34	4
−554	17800	+55	1	−352	15250	−6	11	−128	12600	−15	8
−554	17550	+72	1	−316	15600	+10	10	−119	12500	+66	1
−554	16900	+100	1	−316	16650	+44	2	−119	11750	+93	1
−554	16100	+120	1	−316	16200	+63	1	−119	10950	+120	1
−536	18800	−14	8	−307	14150	−10	10	−109	14600	+25	5
−525	19300	+60	1	−239	14300	−3	12	−108	12100	+8	10
−525	18400	+78	1	−238	8850	+80	1	−105	10500	+66	1
−500	14950	+77	1	−238	8300	+100	1	−105	8900	+126	1
−482	17300	−10	10	−238	7800	+120	1	−105	12900	−50	2
−464	15600	+20	6	−225	17600	+52	2	−105	11850	−29	4
−423	16700	+50	2	−225	17550	+69	1	−105	12950	−8	10
−423	17050	+74	1	−225	19000	+79	1	−104	11750	−26	5
−423	17050	+100	1	−225	19400	+94	1	−104	12050	−12	10
−420	15400	+19	6	−214	14200	+30	4	−95	13200	+57	1
−409	22400	+68	1	−214	12900	+51	2	−80	8900	+60	1
−409	21600	+95	1	−214	14050	+67	1	−79	11500	−40	3
−409	19400	+128	1	−214	13300	+86	1	−79	10850	−16	8
−408	15200	+8	10	−211	21300	+28	0	−79	12100	+30	4
−407	15250	+15	8	−193	13600	−12	10	−72	12900	−37	3
−406	15000	−48	2	−189	10800	−30	4	−72	12150	−17	8
−406	12500	−27?	0	−189	11300	−10	10	−66	10100	+23	5
−405	16500	−14	8	−188	10800	−34	3	−65	9500	+13	9
−396	15500	+48	2	−188	11350	−18	6	−65	9900	+32	3
−396	12900	+75	1	−162	9500	−85	1				
		continued above . . .				*continued above . . .*					

ΔT(s) : Correction to the Earth's clock in seconds
Degree : Babylonian clock interval in degrees. $1^\circ = 4$ minutes of time
Weight : Weight assigned to observation

Table S2. Babylonian lunar eclipses, culmination of ziqpu star

Year	ΔT(s)	Weight	Year	ΔT(s)	Weight	Year	ΔT(s)	Weight
− 225	15500	3	− 149	11400	3	− 119	11900	3
− 193	14000	3	− 142	12750	3	− 104	12000	3
− 189	10400	1	− 135	11800	3	− 95	11600	3
− 182	10100	3	− 134	11900	3	− 93	9800	3
− 177	13200	3	− 133	11500	3	− 86	12000	3
− 162	10400	3	− 128	11350	3	− 80	10350	3
− 159	12700	3	− 122	14000	3	− 79	11300	3
	continued above . . .			*continued above . . .*				

ΔT(s) : Correction to the Earth's clock in seconds
Weight : Weight assigned to observation

Table S3. Babylonian solar eclipses

Year	ΔT(s)	Degree	Weight	Year	ΔT(s)	Degree	Weight	Year	ΔT(s)	Degree	Weight
− 356	15600	− 76	1	− 189	12900	+30	4	− 132	11500	− 50	2
− 321	14200	− 3	12	− 189	13400	+45	2	− 132	11700	− 30	4
− 280	13300	+ 6	11	− 189	14150	+60	1	− 132	11700	− 12	10
− 280	12950	+ 20	6	− 169	12300	−20	6	− 88	9200	+ 45	2
− 253	11400	− 56	1	− 169	12200	− 8	10	− 88	10100	+ 57	1
− 253	11500	− 44	2	− 135	12600	+24	5	− 88	10900	+ 69	1
− 253	11650	− 33	4	− 135	12100	+42	2	− 9	9900	− 90	1
− 248	13800	+ 90	1	− 135	12300	+59	1	− 9	9300	− 67	1
		continued above . . .				*continued above . . .*		− 9	8100	− 42	2

ΔT(s) : Correction to the Earth's clock in seconds
Degree : Babylonian clock interval in degrees. $1^\circ = 4$ minutes of time
Weight : Weight assigned to observation

Table S4. Babylonian lunar eclipses in the Almagest

Year	ΔT(s) Limits Lower bound	Upper bound	Note
− 382	15700	[21450]	Short duration – limits defined by first contact and Moon set eclipsed.
− 719	· · ·	21670	First contact after moonrise.
− 381	· · ·	17850	First contact after sunset.

Discrete results from timing of contacts or maximal phase

Year	ΔT(s)	Measurement	Weight
− 720	21900	began $1\frac{1}{2}$ equal hours after moonrise	3
− 719	19050	max exactly at midnight	1
− 620	17800	began 11 seasonal hours after sunset	1
− 522	19250	max 1 equal hour before midnight	1
− 501	18950	max $6\frac{1}{3}$ equal hours after sunset	1
− 490	13850	max $\frac{1}{2}$ seasonal hour before midnight	1
− 382	17800	began $\frac{1}{2}$ seasonal hour before sunrise	3
− 381	16400	began $\frac{1}{2}$ seasonal hour after sunset	3
− 381	16050	began $2\frac{1}{2}$ seasonal hours before midnight	1

ΔT(s) : Correction to the Earth's clock in seconds
Upper/lower bound : Limits on ΔT, dependent on the eclipse phenomena
[ ] : Uncritical limit
· · · : Redundant limit
Measurement : Comment on how the observations was timed

rspa.royalsocietypublishing.org Proc R Soc A 2016.0404 (S)

Table S5. Chinese timed lunar eclipses

Year	ΔT(s)	Weight	Year	ΔT(s)	Weight	Year	ΔT(s)	Weight
434	1750	0	1069	750	2	1099	600	2
434	2800	0	1071	450	2	1099	250	2
437	7400	1	1071	300	2	1106	200	2
437	6000	1	1071	900	2	1106	950	2
437	6550	1	1073	1000	2	1168	0	2
440	6200	1	1073	1400	2	1168	100	2
440	8100	1	1073	750	2	1270	− 400	2
543	4600	1	1074	1600	2	1270	400	2
585	5900	1	1074	500	2	1270	700	2
585	5600	1	1078	300	2	1272	700	2
585	7100	1	1078	700	2	1272	100	2
592	5850	1	1081	1350	2	1272	100	2
593	4700	1	1082	1650	2	1277	450	2
595	5400	1	1082	1150	2	1277	− 50	2
595	4000	1	1082	1200	2	1277	900	2
595	3500	1	1085	150	2	1277	200	2
596	4200	1	1085	250	2	1279	450	2
596	4800	1	1088	650	2	1279	− 150	2
948	4450	0	1088	150	2	1279	− 400	2
1052	2100	2	1089	1100	2	1279	700	2
1063	3150	2	1092	700	2	1279	900	2
1069	2650	2	1099	1150	2	1279	500	2
1069	1700	2	1099	1050	2	1280	1100	2
	continued above . . .			*continued above . . .*				

ΔT(s) : Correction to the Earth's clock in seconds
Weight : Weight assigned to observation

Table S6. Chinese timed solar eclipses

Year	ΔT(s)	Weight	Year	ΔT(s)	Weight	Year	ΔT(s)	Weight
586	3700	1	1053	2450	2	1173	650	2
586	6700	1	1054	1900	2	1173	1950	2
586	4900	1	1059	2400	2	1173	1400	2
594	6300	1	1066	1650	2	1183	800	2
680	2450	1	1068	1600	2	1195	850	2
691	1000	0	1068	1200	2	1202	700	2
702	2350	1	1068	850	2	1202	1500	2
761	2300	1	1069	500	2	1216	400	2
761	3600	1	1080	0	2	1243	350	2
761	2650	1	1094	1200	2	1245	1350	2
937	2100	2	1094	2650	2	1260	350	2
1040	2850	2	1107	1050	2	1277	300	2
1046	2100	2	1107	1700	2	1277	500	2
1052	2900	2	1107	1150	2	1277	600	2
	continued above . . .			*continued above . . .*				

ΔT(s) : Correction to the Earth's clock in seconds
Weight : Weight assigned to observation

Table S7. Greek timed lunar and solar eclipses

Year	ΔT(s)	Weight	Year	ΔT(s)	Weight	Year	ΔT(s)	Weight
− 200	12400	3	− 173	11000	3	364	6800	3
− 199	11800	3	− 173	10750	3	364	6100	3
− 199	12650	3	− 140	8550	0	364	6000	3
− 199	13550	3	125	10350	3			
	continued above . . .			*continued above . . .*				

ΔT(s) : Correction to the Earth's clock in seconds
Weight : Weight assigned to observation

rspa.royalsocietypublishing.org Proc R Soc A 2016.0404 (S)

Table S8. Arab timed solar eclipses

Year	ΔT(s)	Weight	Year	ΔT(s)	Weight	Year	ΔT(s)	Weight
829	850	0	901	1550	4	978	2000	4
829	2250	4	923	1900	4	979	1450	4
866	2200	4	923	1600	4	985	1500	4
866	2500	4	928	1800	4	985	750	4
866	2450	4	977	1800	4	993	2000	4
891	1650	4	977	2000	4	993	1400	4
901	1700	4	978	1250	4	1004	1450	4
	continued above . . .			*continued above . . .*		1004	1150	4

ΔT(s) : Correction to the Earth's clock in seconds
Weight : Weight assigned to observation

Table S9. Arab timed lunar eclipses

Year	ΔT(s)	Weight	Year	ΔT(s)	Weight	Year	ΔT(s)	Weight
854	3150	1	933	2350	1	1001	450	1
854	2500	1	979	1500	1	1002	1750	1
854	1300	1	979	1900	1	1002	1950	1
856	2300	1	979	1300	1	1003	1450	1
883	900	1	980	2100	1	1003	650	1
901	400	1	981	2000	1	1004	2600	1
923	1200	1	981	2350	1	1019	1900	1
923	2000	1	981	1500	1	1019	1700	1
925	2700	1	983	1200	1	1019	1750	1
925	2300	1	986	800	1	1019	1550	1
927	2900	1	990	3500	1			
	continued above . . .			*continued above . . .*				

ΔT(s) : Correction to the Earth's clock in seconds
Weight : Weight assigned to observation

Table S10. Untimed total and annular solar eclipses

Year	ΔT(s) Limits Upper bound	ΔT(s) Limits Lower bound	Region	Year	ΔT(s) Limits Upper bound	ΔT(s) Limits Lower bound	Region	Year	ΔT(s) Limits Upper bound	ΔT(s) Limits Lower bound	Region
− 708	21100	20160	China	873	3740	1820	Arab	1221	960	− 9600	China
− 600	21900	21120	China	912	2580	840	Arab	1239	1420	− 500	Europe
− 548	21640	16160	China	968	2620	1560	Europe	1241	1380	620	Europe
− 309	17160	13300	Greek	975	4480	1160	Japan	1267	800	− 900	Europe
− 180	12700	11780	China	1061	2140	800	Arab	1275	1280	− 740	China
− 135	12140	11220	Babylon	1124	2700	980	Europe	1292	1820	− 60	China
− 9	10720	8640	Babylon	1133	1160	520	Europe	1406	740	160	Europe
71	9440	9830	Greece	1147	1160	280	Europe	1415	680	− 800	Europe
454	7900	6120	China	1176	1600	600	Arab	1431	680	− 200	Europe
761	3260	1700	China	1178	1900	780	Europe	1485	780	− 5500	Europe
840	6800	1940	Europe	1185	10500	− 2100	Europe	1560	210	− 480	Europe
		continued above . . .				*continued above . . .*		1567	165	145	Europe

ΔT(s) : Correction to the Earth's clock in seconds
Upper/lower bound : Limits on ΔT, dependent on the eclipse phenomena
Region : Where the eclipse was observed

Table S11. Untimed partial solar eclipses

Year	ΔT(s) Limits Lower bound	Upper bound	Region	Year	ΔT(s) Limits Lower bound	Upper bound	Region	Year	ΔT(s) Limits Lower bound	Upper bound	Region
− 430	12620	[11760]	Greek	360	[9420]	7100	China	1135	[3800]	1840	China
− 393	14280	[12640]	Greek	494	[6600]	5980	China	1147	· · ·	1660	Europe
− 241	12260	[11680]	Babylon	702	2720	[1440]	China	1178	· · ·	1140	Europe
− 187	[14100]	13780	China	729	1180	[400]	China	1330	[1220]	900	Europe
− 79	8460	[8160]	China	822	- - -	4000	China	1361	[1760]	500	China
− 27	9480	[8050]	China	1004	1980	1760	Arab	1605	- - -	1060	Europe
120	8980	8140	China	1133	· · ·	1440	Europe				
		continued above ...				*continued above ...*		· · · - - - redundant (see section 3e)			

ΔT(s) : Correction to the Earth's clock in seconds
Upper/lower bound : Limits on ΔT, dependent on the eclipse phenomena
[ ] : Uncritical limit
· · · : Redundant limit
- - - : Only one viable limit
Region : Where the eclipse was observed

Table S12. Untimed solar eclipses rose or set eclipsed

Year	ΔT(s) Limits Lower bound	Upper bound	Region	Year	ΔT(s) Limits Lower bound	Upper bound	Region	Year	ΔT(s) Limits Lower bound	Upper bound	Region
− 321	13400	[18000]	Babylon	− 280	12350	[17250]	Babylon	− 240	12800	[34000]	Babylon

ΔT(s) : Correction to the Earth's clock in seconds
Upper/lower bound : Limits on ΔT, dependent on the eclipse phenomena
[ ] : Uncritical limit
Region : Where the eclipse was observed

Table S13. Untimed lunar eclipses rose or set eclipsed

Year	ΔT(s) Limits Lower bound	Upper bound	Region	Year	ΔT(s) Limits Lower bound	Upper bound	Region	Year	ΔT(s) Limits Lower bound	Upper bound	Region
− 701	18450	· · ·	Babylon	− 363	· · ·	15550	Babylon	− 189	· · ·	13050	Babylon
− 694	· · ·	19950	Babylon	− 239	· · ·	13750	Babylon	− 98	10100	· · ·	Babylon
· · · redundant								− 79	· · ·	11850	Babylon

ΔT(s) : Correction to the Earth's clock in seconds
Upper/lower bound : Limits on ΔT, dependent on the eclipse phenomena
· · · : Redundant limit
Region : Where the eclipse was observed

Table S14. Untimed lunar eclipses: estimates of degree of obscuration at rising or setting

Year	ΔT(s)	Region	Year	ΔT(s)	Region	Year	ΔT(s)	Region
− 536	18150	Babylon	− 352	14500	Babylon	− 98	12800	Babylon
− 363	15250	Babylon	− 189	12200	Babylon			

ΔT(s) : Correction to the Earth's clock in seconds
Region : Where the eclipse was observed

rspa.royalsocietypublishing.org Proc R Soc A 2016.0404 (S)

Table S15. Polynomial coefficients for ΔT from −720.0 to 2016.0

Row	Years		Polynomial coefficients			
i	K_i	K_{i+1}	a_0	a_1	a_2	a_3
1	−720.0	400.0	20550.593	−21268.478	11863.418	−4541.129
2	400.0	1000.0	6604.404	−5981.266	−505.093	1349.609
3	1000.0	1500.0	1467.654	−2452.187	2460.927	−1183.759
4	1500.0	1600.0	292.635	−216.322	−43.614	56.681
5	1600.0	1650.0	89.380	−66.754	31.607	−10.497
6	1650.0	1720.0	43.736	−49.043	0.227	15.811
7	1720.0	1800.0	10.730	−1.321	62.250	−52.946
8	1800.0	1810.0	18.714	−4.457	−1.509	2.507
9	1810.0	1820.0	15.255	0.046	6.012	−4.634
10	1820.0	1830.0	16.679	−1.831	−7.889	3.799
11	1830.0	1840.0	10.758	−6.211	3.509	−0.388
12	1840.0	1850.0	7.668	−0.357	2.345	−0.338
13	1850.0	1855.0	9.317	1.659	0.332	−0.932
14	1855.0	1860.0	10.376	−0.472	−2.463	1.596
15	1860.0	1865.0	9.038	−0.610	2.325	−2.497
16	1865.0	1870.0	8.256	−3.450	−5.166	2.729
17	1870.0	1875.0	2.369	−5.596	3.020	−0.919
18	1875.0	1880.0	−1.126	−2.312	0.264	−0.037
19	1880.0	1885.0	−3.211	−1.894	0.154	0.562
20	1885.0	1890.0	−4.388	0.101	1.841	−1.438
21	1890.0	1895.0	−3.884	−0.531	−2.473	1.870
22	1895.0	1900.0	−5.017	0.134	3.138	−0.232
23	1900.0	1905.0	−1.977	5.715	2.443	−1.257
24	1905.0	1910.0	4.923	6.828	−1.329	0.720
25	1910.0	1915.0	11.142	6.330	0.831	−0.825
26	1915.0	1920.0	17.479	5.518	−1.643	0.262
27	1920.0	1925.0	21.617	3.020	−0.856	0.008
28	1925.0	1930.0	23.789	1.333	−0.831	0.127
29	1930.0	1935.0	24.418	0.052	−0.449	0.142
30	1935.0	1940.0	24.164	−0.419	−0.022	0.702
31	1940.0	1945.0	24.426	1.645	2.086	−1.106
32	1945.0	1950.0	27.050	2.499	−1.232	0.614
33	1950.0	1953.0	28.932	1.127	0.220	−0.277
34	1953.0	1956.0	30.002	0.737	−0.610	0.631
35	1956.0	1959.0	30.760	1.409	1.282	−0.799
36	1959.0	1962.0	32.652	1.577	−1.115	0.507
37	1962.0	1965.0	33.621	0.868	0.406	0.199
38	1965.0	1968.0	35.093	2.275	1.002	−0.414
39	1968.0	1971.0	37.956	3.035	−0.242	0.202
40	1971.0	1974.0	40.951	3.157	0.364	−0.229
41	1974.0	1977.0	44.244	3.198	−0.323	0.172
42	1977.0	1980.0	47.291	3.069	0.193	−0.192
43	1980.0	1983.0	50.361	2.878	−0.384	0.081
44	1983.0	1986.0	52.936	2.354	−0.140	−0.166
45	1986.0	1989.0	54.984	1.577	−0.637	0.448
46	1989.0	1992.0	56.373	1.649	0.709	−0.277
47	1992.0	1995.0	58.453	2.235	−0.122	0.111
48	1995.0	1998.0	60.677	2.324	0.212	−0.315
49	1998.0	2001.0	62.899	1.804	−0.732	0.112
50	2001.0	2004.0	64.082	0.675	−0.396	0.193
51	2004.0	2007.0	64.555	0.463	0.184	−0.008
52	2007.0	2010.0	65.194	0.809	0.161	−0.101
53	2010.0	2013.0	66.063	0.828	−0.142	0.168
54	2013.0	2016.0	66.917	1.046	0.360	−0.282

For the year and fraction (Y) extract the coefficients a_0, a_1, a_2, a_3 from row i, where $K_i \leq Y \leq K_{i+1}$. Then form $t = (Y - K_i)/(K_{i+1} - K_i)$ and thus calculate $\Delta\mathrm{T} = a_0 + a_1\,t + a_2\,t^2 + a_3\,t^3$ seconds.
